# Preparation and Characterization of Carbon Quantum Dots (CQD) and CuFe_2_O_4_–CQD Composite Materials for Photo and Electrochemical Applications

**DOI:** 10.1002/gch2.202500044

**Published:** 2025-06-09

**Authors:** Esakkimuthu Shanmugasundaram, Amos Ravi, Nithesh Kumar Krishnan, Kannan Vellaisamy, Murali Krishnan Mani, Na'il Saleh, Stalin Thambusamy

**Affiliations:** ^1^ Department of Industrial Chemistry Alagappa University Karaikudi Tamil Nadu 630 003 India; ^2^ Department of Materials Science Central University of Tamil Nadu Thiruvarur Tamil Nadu 610 005 India; ^3^ Department of Chemistry Bannari Amman Institute of Technology Sathyamangalam Erode Tamil Nadu 638 401 India; ^4^ Department of Chemistry College of Science United Arab Emirates University P.O. Box 15551 Al Ain UAE

**Keywords:** CQD, CuFe_2_O_4_–CQD, electrochemical performance, fluorescence sensing, supercapacitor

## Abstract

The Carbon quantum dots (CQD) is prepared from ascorbic acid, and the photophysical, structural, and metal sensing behavior of the CQD is investigated in detail. The negatively charged CQD, along with the vibrant functional groups, can absorb the positive charge ferric ion (Fe^3+^) and copper(II) ion or cupric ion (Cu^2+^) ions with the help of electrostatic attractive forces. In this process, the aggregation of CQD around the Fe^3+^ and Cu^2+^ ions results in confirmation that CQD is a fluorescence sensor probe that forms a metal complex, CQD‐Fe^3+^ and CQD‐Cu^2+^. The composite structural and functional group properties are investigated by the different analytical techniques. Moreover, the copper ferrite (CuFe^2^O^4^–CQD) electrochemical performances are evaluated in three and two‐electrode systems by Cyclic voltammetry (CV), Galvanostatic charge‐discharge (GCD), and Electrochemical Impedance Spectroscopy (EIS) techniques. The CuFe^2^O^4^–CQD electrode's specific capacitance value is 410 F g^−1^ at 2 A g^−1^ with 100% capacitance retention after 3000 cycles. Moreover, the methylene blue dye degradation efficiency of CuFe^2^O^4^–CQD is 91% in 120 min. The CuFe^2^O^4^–CQD composite has a synergistic effect between the CQD and CuFe^2^O^4^, which delivers a higher photocatalytic effect because which reduced recombination and enhancing charge transport.

## Graphical abstract

The incorporation of copper and iron metal ions in the carbon quantum dots, enhances their combined use as supercapacitors and biomarkers and optimizes their degradation efficiency.

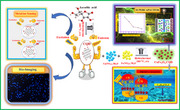

## 1 Introduction

Carbon quantum dots (CQDs) are quasi‐spherical‐shaped nanoparticles whose size is less than 10 nm.^[^ ^1^ ^]^ They have 0D nanostructures that possess graphitic sp^2^ or sp^3^ carbon hybridization. CQDs have superior electrical and optical properties, as well as it has some unique properties such as good biocompatibility, low toxicity, and high water solubility.^[^ ^2^ ^]^ These properties enable CQDs to be used in various environmental and energy applications like sensors,^[^ ^3^ ^]^ bio‐imaging,^[^ ^4^ ^]^ photodynamic therapy,^[^ ^5^ ^]^ solar cells,^[^ ^6^ ^]^ supercapacitors,^[^ ^7^ ^]^ and photocatalytic applications.^[^ ^8^ ^]^ Particularly, CQDs have received much more attention in the field of fluorescence metal sensing^[^ ^9^ ^]^ due to their sufficient optical (absorbance and emission) properties. CQDs have different types of core structure, which is sp^2^ hybridized carbon or mixed sp^2^ and sp^3^. The functional groups are bound to the metal ion and involve a fluorescence off‐on sensing mechanism.^[^ ^10^ ^]^ Different type of CQDs derived from various sources and methods that detect Fe^3+^, Fe^2+^, Hg^2+^, and Cu^2+^ depends upon the functional group present in the CQDs moiety. For instance, CQD from citric acid‐diethylenetriamine^[^ ^11^ ^]^ source detects Hg^2+^ and I^−^, whereas citric acid‐5,10,15,20‐tetrakis(4‐sulfophenyl) porphyrin (TSPP)–CQD^[^ ^12^ ^]^ detects Cu^2+^. Ag/Au doped CQD^[^ ^13^ ^]^ have high emission properties that sense the Cu^2+^ and Hg^2+^ ions. Boron‐doped CQD^[^ ^14^ ^]^ is derived from naphthalene‐1‐boronic acid and catechol, which detects Mg^2+^, whereas the N–CQD from glucose and ammonia^[^ ^15^ ^]^ detects Cr^4+^.

Moreover, the CQDs are used in bio‐imaging applications owing to their various color emission based on their quantum dot sizes.^[^ ^16^ ^]^ CQDs have a hydrophobic nature so they easily disperse in all biological media. For instance, cabbage‐derived CQD shows low cytotoxicity and emits three types of emission colors in the treated cells.^[^ ^17^ ^]^ CQD from Phoenix dactylifera leaf shows excellent biocompatibility at 200 µg mL^−1^ and outstanding bio‐imaging performance in the labelled cells.^[^ ^18^ ^]^

Keeping in mind the above effective results of CQD in sensing and bio‐imaging applications, here, the CQD was prepared from ascorbic acid and analyzed for metal ion detection and bio‐imaging behavior. In our previous works, the CQD was a light conversion material that was doped with conducting polymers such as polyaniline^[^ ^19^, ^20^ ^]^ and polythiophene^[^ ^21^ ^]^ by electro‐polymerization for organic solar cell application. Due to the superior absorption and emission properties, in this paper, the CQD is involved in metal sensing and bio‐imaging applications. The prepared CQD can detect Fe^3+^ and Cu^2+^ with the help of the FRET mechanism. The result confirmed that the CQD has more affinity to bind the Fe^3+^ and Cu^2+^ ions than other metal ions that property. Moreover, the CQD is an efficient bio‐imaging agent for MDA‐MB‐231 cancer cells.

The sensing behavior is boosting our interest in preparing CuFe_2_O_4_–CQD composite for supercapacitor and photo‐catalytic dye degradation application because the metal sensing results confirm that the CQD can effectively interact with the Fe^3+^ and Cu^2+^ ions. Basically metal oxides have received much more attention in the field of electrocatalytic reduction,^[^ ^22^, ^23^, ^24^ ^]^ water‐splitting,^[^ ^25^ ^]^ and energy storage applications.^[^ ^26^ ^]^ CuFe_2_O_4_ (copper ferrite) is a metal complex that has an inverse spinel structure (AB_2_X_4_).^[^ ^27^ ^]^ The CuFe_2_O_4_ structure consists of two cations, which are Fe^3+^ (A‐tetrahedral) and Cu^2+^ (B‐octahedral).^[^ ^28^ ^]^ Xiai Zhang et al. reported that magnetic CuFe_2_O_4_ exhibits superior capacitance and cyclic performance, which is 960.73 F g^−1^ at 1 A g^−1^, and a capacitance retention is 57.1%.^[^ ^29^ ^]^ Zhu et al. synthesized the CuFe_2_O_4_ nanospheres, delivering 334 F g^−1^, and the capacitance retention is 88%.^[^ ^30^ ^]^ Although CuFe_2_O_4_ is a potential material for capacitor applications, the lower conductivity and poor stability are negative factors for use in commercial applications. To overcome these issues, carbon materials like CNF, CNT, and graphene are added to the CuFe_2_O_4_ nanocomposite because they are carbon materials (higher surface area), which improve the insertion–extraction process in the electrolyte ions. For instance, the rGO‐doped CuFe_2_O_4_ delivered 797 F g^−1^,^[^ ^31^ ^]^ and CuFe_2_O_4_–graphene sheet gave 576.6 F g^−1^ capacitance at 1 A g^−1^.^[^ ^32^ ^]^ Electro‐spun carbon/CuFe_2_O_4_ delivered 0.172 Wh kg^−1^ energy and 310.4 W kg^−1^ power density.^[^ ^33^ ^]^

In the photocatalytic dye degradation application, Oliveira et al. reported that the rhodamine b dye degradation efficiency of CuFe_2_O_4_ is 84.30% under visible light irradiation.^[^ ^34^ ^]^ Moreover, CuFe_2_O_4_/GO@biochar degrades 98.9% of malachite green in the presence of visible light.^[^ ^35^ ^]^ CuFe_2_O_4_/g‐C_3_N_4_ destroys 82% propranolol under visible light with the help of peroxydisulfate.^[^ ^36^ ^]^

Therefore, in this work, the prepared CQD (carbon additive material) is doped with CuFe_2_O_4_ to enhance the optoelectrical properties of CuFe_2_O_4_ because the CQD has sufficient optical and electrical properties than carbon and other materials. The prepared CuFe_2_O_4_–CQD was involved in structural, optical, and electrochemical studies. The CuFe_2_O_4_–CQD possesses better capacitance and dye degradation efficiency than CuFe_2_O_4_ owing to the combination of the excellent redox capability of CuFe_2_O_4_ and the good optoelectrical properties of CQD.

## 2 Results and Discussion

### 2.1 Carbon Quantum Dot Characterizations

**Figure 1:**
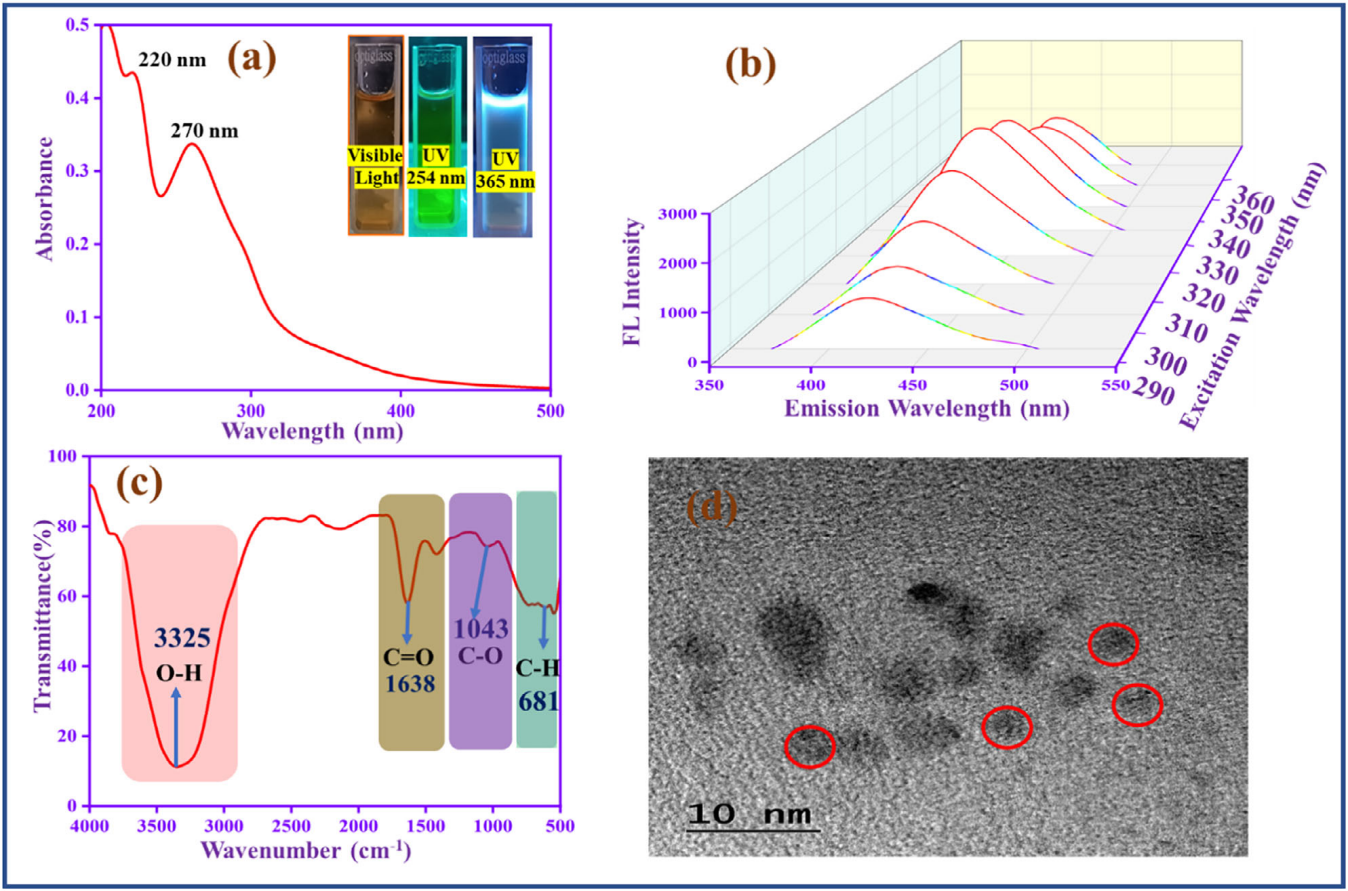
a) UV spectrum, b) fluorescence spectrum (290–360 nm), c) FT‐IR spectrum, d) TEM image of CQD.

UV spectrum of carbon quantum dot (CQD) is shown in **Figure** **1**a, which has two peaks at 220 nm, representing to the C─C and C═C, and 270 nm denotes C═O functional groups. The peaks confirm that this type of functional group is present on the CQD outer surface.^[^ ^37^ ^]^ The emission behavior of CQD in different excitation ranges is depicted in the inset photos.

In addition, to analyze the emission behavior of CQD, involving excitation dependent study (290–360 nm), as shown in Figure 1b. When the excitation is increased, the peak intensity is increased and redshifted. The redshift reason is the π→ π* transition (graphitic sp^2^) of CQD at lower excitation energies. The CQD reaches a higher intensity at 330 nm, then decreases, which confirms the CQD has various emission traps and different‐sized dots.^[^ ^38^ ^]^ FT‐IR spectroscopy (Figure 1c) helps to identify the functional groups in the CQD. The U‐shape peak (3325 cm^−1^) represents the hydroxyl functional group (─OH) present in the CQD. The peaks at 1638 and 1043 cm^−1^ are attributed to the stretching frequency of carbonyl groups (C═O) and (C─O), respectively.^[^ ^39^ ^]^ Moreover, the peak at 675 cm^−1^ corresponds to the C─H functional group. Moreover, Figure 1d shows the TEM image of CQD, which is spherical, and the diameter is 2–10 nm.^[^ ^40^ ^]^ Figure S1 (Supporting Information) exhibits the particle size distribution of CQD, in which the average diameter is 3.7 nm. Figure S2 (Supporting Information) exhibits the zeta potential study of CQD. The CQD negative zeta potential value (−8 mV), which confirms ─COOH, ─OH, and C═O electronegative functional groups are present in the CQD surface.^[^ ^41^ ^]^ Figure S3 (Supporting Information) shows the fluorescence emission stability study of CQD. It displays that the synthesized CQD exhibits stability over 7 days without any changes in the fluorescence emission.

### 2.2 Metal Sensing Study of CQD

#### 2.2.1 Sensing Behavior of CQD by Fluorescence Spectroscopy

The CQD involves a fluorescence selectivity study. The fluorescence spectra of the CQD emission peak are presented at 410 nm. 50 *µ*M of various metal ions in 1 × 10^−6^ m concentration (Al^3+^, Ba^2+^, Fe^3+^, Fe^2+^, Co^2+^, Cu^2+^, Mg^2+^, Mn^2+^, Pb^2+^) are added with 2 mL of CQD solution. In Figure S4 (Supporting Information), except for Fe^3+^ and Cu^2+^, CQD fluorescence intensity does not change when the metal ions are added. When the Fe^3+^ and Cu^2+^ interact with CQD, the fluorescence intensity is decreased. The Fe^3+^ and Cu^2+^ can easily bind to the CQD surface through carboxyl and hydroxy functional groups. The result proves that the Fe^3+^ and Cu^2+^ coordinate with the CQD and involve the frontier resonance energy transfer (FRET) quenching mechanism.^[^ ^42^ ^]^ The CQD is a good sensor probe for Fe^3+^ and Cu^2+^ ions detection by fluorescence turn‐off effect.

#### 2.2.2 Titration Profile of CQD with the Interactions of Fe^3+^ and Cu^2+^ ions

**Figure 2:**
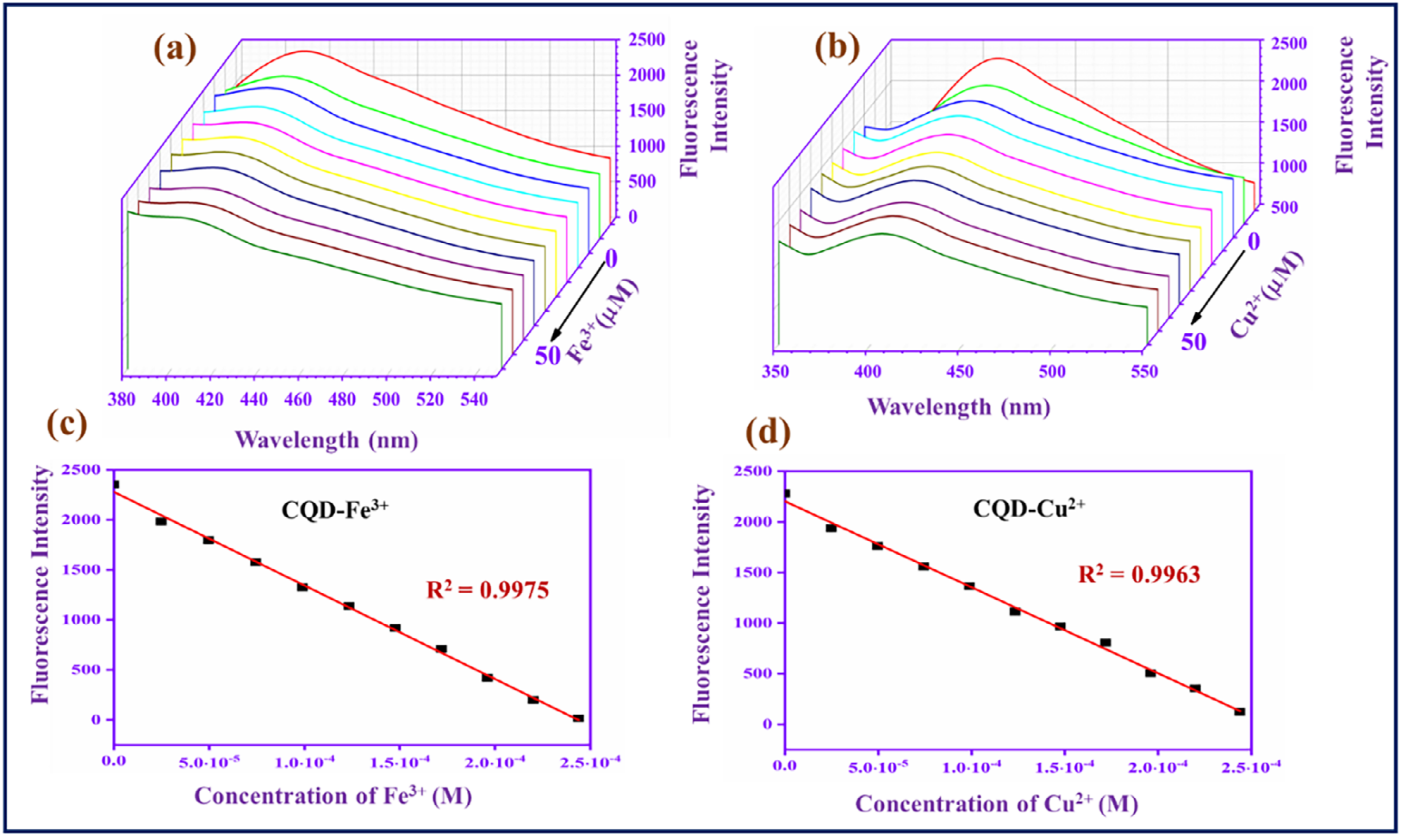
a,b) Fluorescence spectra of different concentrations of Fe^3+^ and Cu^2+^ (0–50 µM), c,d) Linear graph of fluorescence intensity versus concentration of Fe^3+^ and Cu^2+^.

The sensitivity study of the CQD with Fe^3+^ and Cu^2+^ was studied and reported in **Figure** **2**. The concentration range of metal ions is 0–50 *µ*M.

In Figure 2a,b, the CQD fluorescence intensity gradually decreases when the metal ions (Fe^3+^ and Cu^2+^) concentration is increased in the CQD solution. When the concentration reaches 50 *µ*M, the fluorescence intensity of the CQD becomes very weak and reaches a low level. The fluorescence emission of the CQD is quenched by the Fe^3+^ and Cu^2+^ owing to the coordination interaction between the carbonyl and hydroxyl functional groups on the surface of CQD with the corresponding metal ions.^[^ ^43^ ^]^ The coordination complexes CQD–Fe^3+^ and CQD–Cu^2+^ may be contributing to the intramolecular non‐radiative energy transfer, in which the CQD is the energy donor and the Fe^3+^ and Cu^2+^ are acceptors. In Figure 2c and d, for the graph of the intensity against the Fe^3+^ and Cu^2+^ concentration, the linear calibration curve ranges are R^2^ = 0.9975 and R^2^ = 0.9963, respectively. The linear fitting graphs can help to calculate the LOD value, which is 0.36 µM for Fe^3+^ and 0.59 µM for Cu^2+^.

#### 2.2.3 Interference and Real Sample Analysis

CQD probe selectivity study was examined with competitive metals Al^3+^, Ba^2+^, Fe^3+^, Fe^2+^, Co^2+^, Cu^2+^, Mg^2+^, Mn^2+,^ and Pb^2+^ (50 *µ*M), along with Fe^3+^ Figure S5a (Supporting Information) and Cu^2+^ Figure S5b (Supporting Information). It is commonly known that the fluorescence intensity of the stable complex is not affected by other metal ions. In the interference study, the interfering metal ions have a negligible influence CQD–Fe^3+^ and CQD–Cu^2+^ solutions, so that the CQD is selective toward Fe^3+^ and Cu^2+^ sensing probe even in the presence of other metals present in the aqueous medium. The finding helps to understand the selectivity and sensitivity of the CQD probe in detecting Fe^3+^ and Cu^2+^. For more clarification about real‐life applications, the CQD probe involves real sample analysis in which the different water sources, like pond, sea, and tap water, are treated with known amounts of Fe^3+^ and Cu^2+^ ions. The detailed real sample analysis study was tabulated in Tables S1 and S2 (Supporting Information).

The results exhibit the recovery percentages of the various water samples in the concentration ranges from 0 to 10 µM., Notably, the CQD probe recovery rates of tap water (95−98%), pond water (92−97%), seawater (94−98%), for Fe^3+^ and Cu^2+^ ions, respectively. These outcome results confirm that the CQD is an effective sensing probe when applied to real water samples. The high recovery rates suggest that the CQD can sensitively and accurately detect metal ions across different environmental water samples. The above findings confirm that the CQD is a potential sensor probe to detect metal ions in various environmental water resources. Metal sensing performance of CQD in comparison with other reported CQD is shown in Tables S3 (Supporting Information).

#### 2.2.4 Density Functional Theory Study

The geometry optimization of CQD and the complexes CQD–Fe^3+^, and CQD–Cu^2+^ were analyzed by density functional study (DFT) and are shown in Figure S6 (Supporting Information). First, the ground state optimized structure CQD was generated, and metal ions (Fe^3+^ and Cu^2+^) were attached to the core of the carbonyl or hydroxyl functional groups of CQD at a non‐interacting distance. Then, the highly occupied molecular orbital (HOMO) and low unoccupied molecular orbital (LUMO) energy levels of the CQD, CQD–Fe^3+^, and CQD–Cu^2+^ were calculated. The energy gap of CQD is 2.559 eV, whereas the complexes' energy gaps for the CQD–Fe^3+^ is 1.038 eV and CQD–Cu^2+^ is 2.474 eV. These results suggest that the CQD functional group easily binds with the Fe^3+^ and Cu^2+^, changing the energy levels and electronic transitions of the CQD.^[^ ^44^ ^]^

#### 2.2.5 Plausible FRET Transfer Mechanism of CQD with Fe^3+^ and Cu^2+^

The negatively charged CQD, along with the vibrant functional groups, can absorb the positive charge Fe^3+^ and Cu^2+^ ions with the help of electrostatic attractive forces.^[^ ^41^ ^]^ In this process, the aggregation of CQD around the Fe^3+^ and Cu^2+^ ions forms a coordinate bond (CQD‐ Fe^3+^ and CQD‐ Cu^2+^). Then, they involve the charge transfer process between the CQD with Fe^3+^ and Cu^2+^ ions in non‐radiative recombination termed FRET. That is the reason the emission of CQD behavior is reduced when the Fe^3+^ and Cu^2+^ ions are added. The possible mechanism is shown in Figure S7 (Supporting Information).

### 2.3 Bioimaging Study of CQD

**Figure 3:**
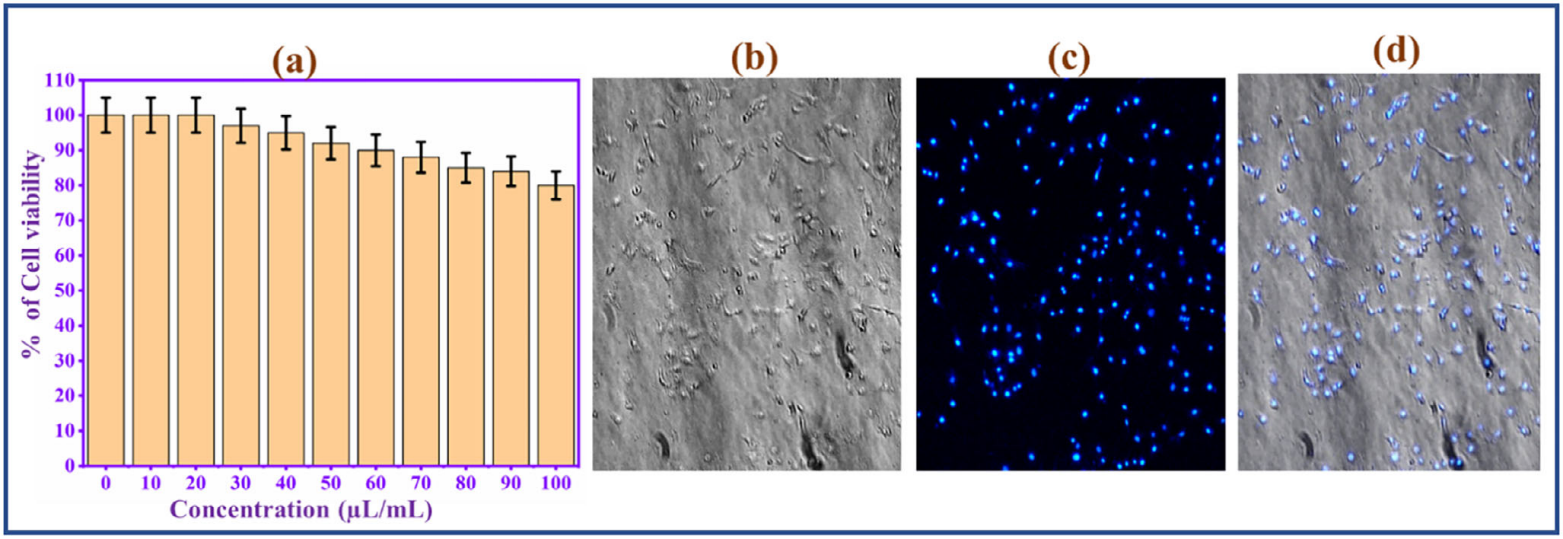
a) Cell viability study of CQD at various concentrations (0–100 µL mL^−1^) in MDA‐MB‐231 cells, Fluorescence microscope images of CQD with MDA‐MB‐231 cells at 360 nm b) Bright Field, c) Fluorescence image, and d) Merged.

Low cytotoxicity and biocompatibility are essential properties of nanomaterials used in bioimaging applications.^[^ ^45^ ^]^ Due to the superior photoluminescence and biocompatibility properties, the prepared CQD involves a fluorescence bio‐imaging study. To analyze the CQD fluorescent probe in bio‐imaging, CQD involves a biocompatibility study with the normal cell lines. MDA‐MB‐231 cells were used as a cancer cell model. The CQD cytotoxicity was evaluated by standard MTT assay (**Figure** **3**a). The cells were incubated with 0–100 µL CQD for 24 h, and the proliferation of the cells was measured. The cell viability of CQD is ≈85% at a high dose concentration (100 µL). This result indicates the CQD has low‐level cytotoxicity and excellent biocompatibility, so it is a potential fluorescence probe for bioimaging applications.

To analyze the imaging behavior of CQD, an in vitro bioimaging study using MDA‐MB‐231 cells was conducted. The cells were incubated with CQD for 8 h. Then, the treated cells were mounted on a fluorescence microscope stage and excitation at 360 nm.^[^ ^46^ ^]^ The cells are brightly illuminated by the blue color. The images are captured with the help of the blue channel of microscopy, as depicted in Figure 3b–d. Interestingly, the morphology of cells is not affected after the bio‐imaging process, which strongly confirms the CQD biocompatibility of the CQD. The results revealed that the CQD could be a sufficient biomarker for bio‐imaging applications.

### 2.4 Characterization Studies of Copper Ferrite ‐Carbon Quantum Dot (CuFe_2_O_4_–CQD) Nanocomposite

#### 2.4.1 Optical and Structural Study

**Figure 4:**
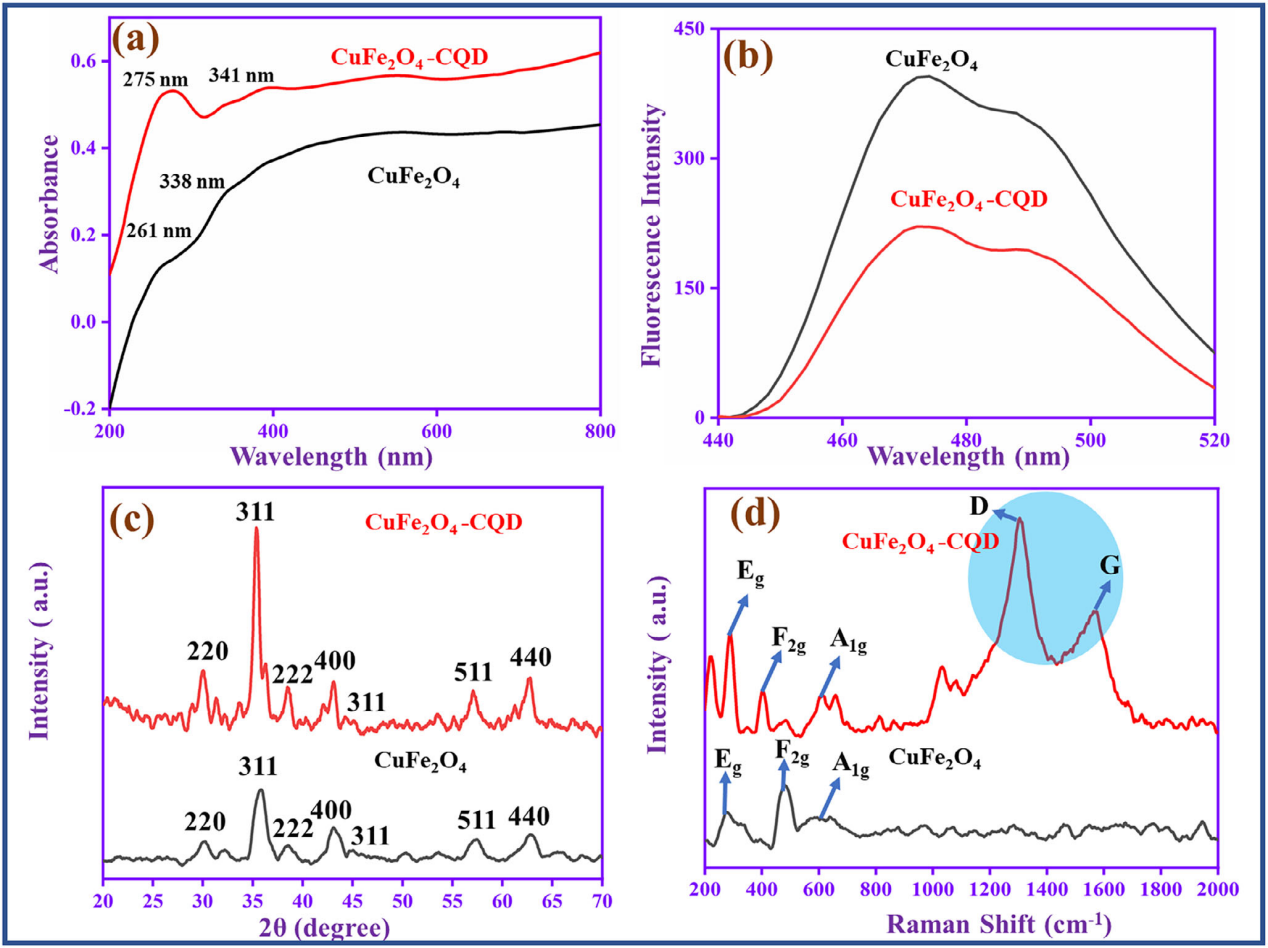
a) UV–vis and b) fluorescence spectra, c) XRD, and d) Raman spectra of CuFe_2_O_4_ and CuFe_2_O_4_–CQD.

The UV–vis spectra of copper ferrite (CuFe_2_O_4_) and CuFe_2_O_4_–CQD are shown in **Figure** **4**a. The CuFe_2_O_4_ has two broad absorption peaks in the range of 261 and 338 nm, which correspond to the charge transfer process of the O^2−^–Cu^2+^ and O^2−^–Fe^3+^ transitions in the spinel structure of CuFe_2_O_4_.^[^ ^47^ ^]^ Compared to CuFe_2_O_4_, the CuFe_2_O_4_–CQD absorption bands are redshifted and appear at 275 and 341 nm due to the interaction between the CQD electronic transition and the copper and iron charge transfer process. For more understanding of the CuFe_2_O_4_ and CuFe_2_O_4_–CQD optical properties, they involve a fluorescence study, as shown in Figure 4b. When CuFe_2_O_4_ is excited at 360 nm, it emits an emission at 470 nm. The emission is owing to the structural defects and impurities present in the nanocrystal. The same range emission peak (470 nm) is also present in the CuFe_2_O_4_–CQD, but the peak intensity is lower than CuFe_2_O_4_. The intensity loss of CuFe_2_O_4_–CQD can be attributed to the electro transfer mechanism from CuFe_2_O_4_ to CQD. The CQD involves non‐radiative energy transfer, that the reason the intensity of the CuFe_2_O_4_–CQD is lost. The intensity loss reveals that the CuFe_2_O_4_–CQD highly reduces the electron‐hole pair recombination.^[^ ^48^ ^]^ The recombination loss can produce a greater number of free electrons during electro and photochemical reactions. The prepared CuFe_2_O_4_ and CuFe_2_O_4_–CQD chemical bond vibrations, confirmed based on infrared radiation by the FT‐IR spectra, are shown in Figure S8 (Supporting Information). The CuFe_2_O_4_ has a broad vibration band presented at 3428 cm^−1^ for the stretching vibration of O─H. Meanwhile, the peak at 1653 cm^−1^ is for the bending vibration of O─H. The CuFe_2_O_4_ metal oxide vibration peaks are located at 770 and 557 cm^−1^, corresponding to Fe─O and Cu─O vibrations, respectively. Same types of peaks such as O─H stretching (3442 cm^−1^), O─H bending (1664 cm^−1^), Fe─O stretching (685 cm^−1^), and Cu─O stretching (540 cm^−1^) also presented in the CuFe_2_O_4_–CQD but the peak is shifted their position due to the interaction of CQD's with the copper ferrite chemical bonds. The CQD's hydroxyl group interacts with the CuFe_2_O_4_ metal surface and creates a hydrogen bond, which can change the vibration range the reason in the peaks being shifted the position. This result suggests that the CQD successfully grafted with CuFe_2_O_4_ in the CuFe_2_O_4_–CQD.^[^ ^49^ ^]^

Figure 4c is the XRD spectra of the prepared materials. The CuFe_2_O_4_ diffraction peaks are matched with the JCPDS 01‐077‐0010 (standard XRD data). The XRD peaks at 2θ = 30.32, 36.00, 38.46, 43.13, 46.22, 57.09, and 62.91° corresponding to the planes (220), (311), (222), (400), (311), (511), and (440) are the crystal planes CuFe_2_O_4_ in cubic spinel structure nature.^[^ ^30^ ^]^ The same type of planes is presented in the CuFe_2_O_4_–CQD, however, the position of the peaks is slightly shifted and located at 2θ = 31.49, 33.22, 35.67, 45.56, 49.64, 56.27, and 63.73° due to the interaction of CQD with CuFe_2_O_4_. There is no extra peak presented in the CuFe_2_O_4_–CQD composite that denotes the CQD does not change the crystal structure of the CuFe_2_O_4_.^[^ ^50^ ^]^

Raman spectroscopy is an effective tool to analyze the fundamental vibration of CuFe_2_O_4_ and CuFe_2_O_4_–CQD molecules, which is shown in Figure 4d. The CuFe_2_O_4_ has three vibration modes present at 280 cm^−1^ (E_g_), 425 cm^−1^ (F_2g_), and 620 cm^−1^ (A_1g_), denoting the tetragonal crystalline phase of CuFe_2_O_4_. The same peaks 283 cm^−1^ (E_g_), 420 cm^−1^ (F_2g_), and 627 cm^−1^ (A_1g_) are presented in the CuFe_2_O_4_–CQD, whereas two additional peaks are presented at 1360 and 1590 cm^−1^, corresponding to the D and G vibrations of CQD. The result confirms that the CQD successfully binds with CuFe_2_O_4_ in the CuFe_2_O_4_–CQD.^[^ ^51^ ^]^

#### 2.4.2 XPS Analysis

**Figure 5:**
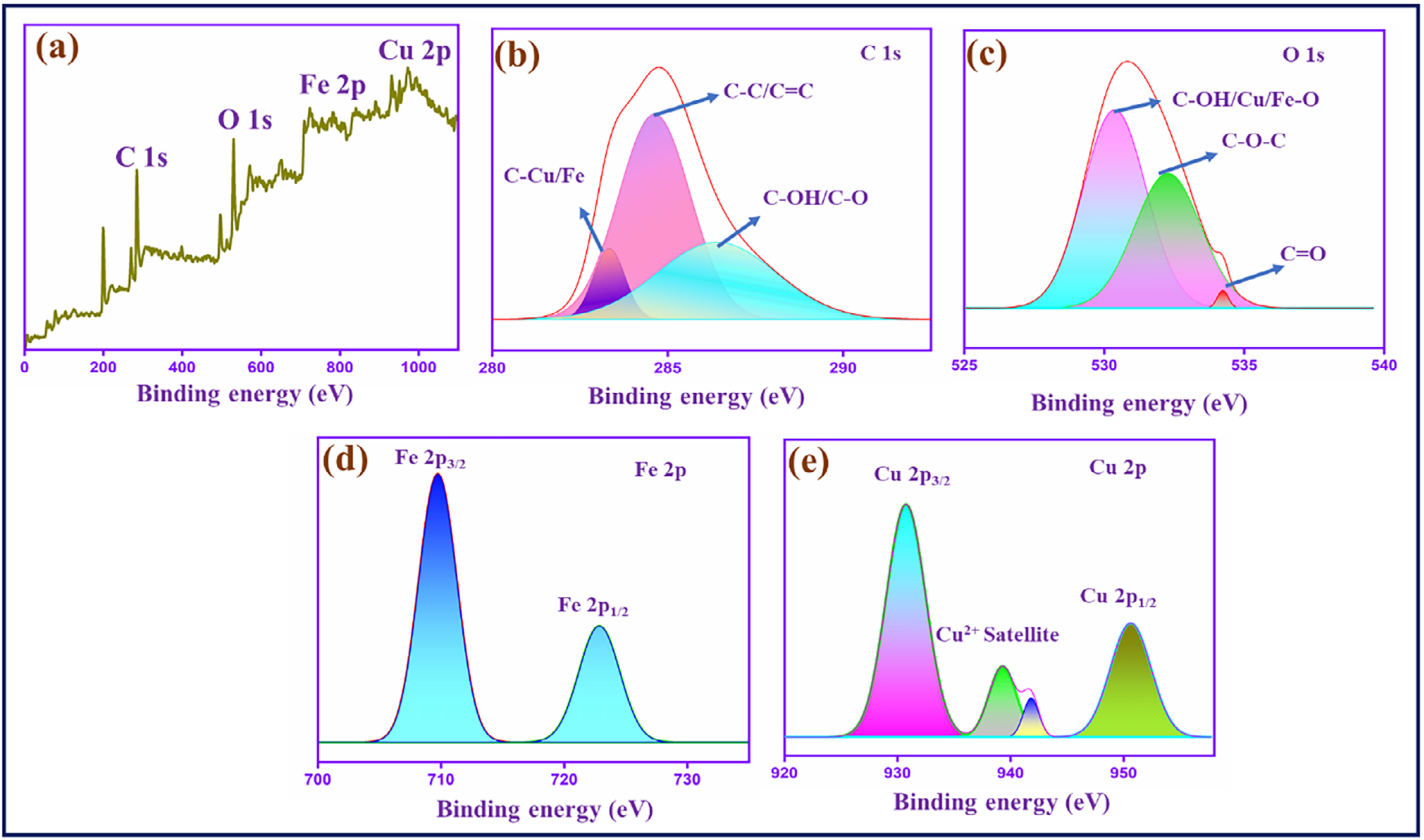
XPS deconvoluted spectra of CuFe_2_O_4_–CQD a) Survey scan, b) C 1s, c) O 1s, and d) Fe 2p e) Cu 2p.

In **Figure** **5**a, the XPS survey spectrum exhibited the carbon (C 1s), oxygen (O 1s), iron (Fe 2p), and copper (Cu 2p) elements presented in the CuFe_2_O_4_–CQD composite.^[^ ^52^, ^53^, ^54^ ^]^ The oxygen and carbon peaks are confirmed by the CQD carbon and oxygen functional groups presented in the CuFe_2_O_4_. In C 1s spectra Figure 5b, the three peaks observed at 283.34, 284.62, and 286.25 eV binding energies are assigned to the functional groups of C─Cu/Fe, C─C/C═C, and C─OH/C─O, respectively.^[^ ^55^ ^]^ This type of chemical bond enhances the ion absorption ability (K^+^ and OH^−^), which promotes the overall electrochemical performance of the prepared electrode materials in the electrochemical cell.^[^ ^56^ ^]^ The O 1s deconvolution spectrum Figure 5c, exhibits three peaks at 530.34, 532.20, and 534.21 eV corresponding to the bonds of C─OH or Cu/Fe─O, C─O─C, and C═O.^[^ ^57^ ^]^ The peaks confirm that CuFe_2_O_4_ can bind to the oxygen‐containing functional groups of CQD. Moreover, the binding energies of the Fe 2p Figure 5d, and copper Cu 2p Figure 5e, ions were found at 709.82 and 930.81 eV. The peaks imply that the iron (Fe^3+^) and copper (Cu^2+^) ions are present in the CuFe_2_O_4_–CQD composite. The Fe 2p^3/2^ and Fe 2p^1/2^ peaks are presented at 709.82 and 723.06 eV.^[^ ^58^ ^]^ Meanwhile, the Cu 2p spectrum reveals that the binding energy of Cu 2p^3/2^ is 930.81 eV and 2p^1/2^ is 950.49 eV.^[^ ^59^ ^]^ The satellite peaks appeared at 939.44 and 941.65 eV. These peaks confirm that the copper ions are in a +2‐oxidation state in the composite. The result suggests that the central atoms Fe^3+^ and Cu^2+^ are linked with the carbon and oxygen atoms, which can enhance the ion storage and electron transport during electrochemical reaction.

#### 2.4.3 Scanning Electron Microscopy Analysis

**Figure 6:**
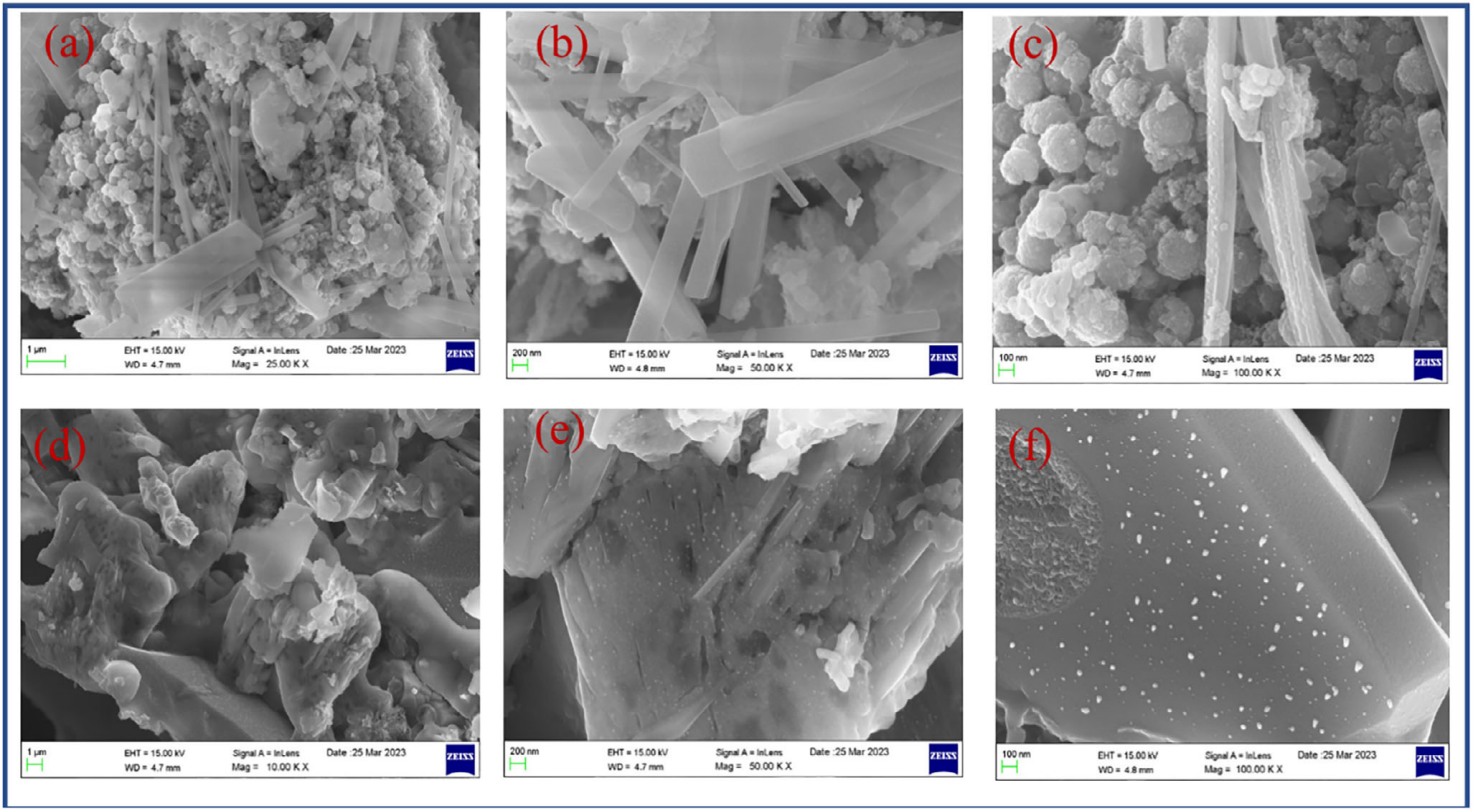
SEM images of a–c) CuFe_2_O_4_, d–f) CuFe_2_O_4_–CQD.

CuFe_2_O_4_ and CuFe_2_O_4_–CQD surface morphology are investigated through scanning electron microscopy (SEM). The CuFe_2_O_4_ SEM images show the nanosphere^[^ ^60^ ^]^ and nanorods structure, which are shown in **Figure** **6**a–c. The nanosphere and nanorods are covered by the carbon sheets in the CuFe_2_O_4_–CQD composite, as shown in Figure 6d–f, For instance, the nanorods of the CuFe_2_O_4_–CQD surface in Figure 6f have white dots corresponding to the CQD, which confirms that the CuFe_2_O_4_ is covered by the CQD.^[^ ^61^ ^]^ The nanorods possess higher surface area for their compact volume, allowing higher active sites which can store more ions and absorb more dye molecules, that properties more beneficial for supercapacitor and photocatalytic applications. Meanwhile, the nanosphere structure has a higher mechanical and electrochemical stability nature that ensures the stability performance of the CuFe_2_O_4_–CQD during the dye degradation process and charge–discharge cycles. The surface interaction of CQD with CuFe_2_O_4_ helps to improve the electrode‐electrolyte interaction, improving the electrochemical performance of the CuFe_2_O_4_–CQD composite.

#### 2.4.4 EDX Analysis

EDX spectra of CuFe_2_O_4_ and CuFe_2_O_4_–CQD help to identify the types of elements in the composites, as shown in Figure S9 (Supporting Information). The quantitative analysis of the CuFe_2_O_4_ EDX spectrum Figure S9a (Supporting Information) exhibits the O, Fe, and Cu weight percentages are 46.13%, 45.81%, and 8.06%, respectively. In CuFe_2_O_4_–CQD Figure S9b (Supporting Information), the O, Fe, and Cu element's weight percentages are 36.20%, 55.81%, and 3.60%. Moreover, 4.39% of carbon is present in the CuFe_2_O_4_–CQD EDS spectrum.^[^ ^62^ ^]^ The result can be concluded that the CQD is present in the CuFe_2_O_4_–CQD composite. There are no additional peaks presented in the composite whose results highlight the purity of the prepared CuFe_2_O_4_ and CuFe_2_O_4_–CQD composites.

#### 2.4.5 Transmittance Electron Microscopy Analysis

Figure S10 (Supporting Information) depicts the morphology and the size of the CuFe_2_O_4_–CQD was characterized by transmission electron microscopy (TEM). Figure S10a–f (Supporting Information) images exhibit the micro/nano sphere‐like structure of the composite.^[^ ^63^ ^]^ Moreover, Figure S10g (Supporting Information) shows the lattice‐resolved HR‐TEM image of the CuFe_2_O_4_–CQD nanocrystal. The interplanar space is 0.22 nm, corresponding to the (311) crystal planes in the cubic spinel structure.^[^ ^64^ ^]^ Additionally, the selected area electron diffraction (SAED) in Figure S10h (Supporting Information) exhibits the pattern of the CuFe_2_O_4_–CQD that gives the crystalline structure information. The SAED pattern contains the diffraction rings owing to their (220), (311), (400), and (511) reflections of cubic spinel spherical Cu.^[^ ^65^ ^]^

#### 2.4.6 Surface Area Analysis

The surface area of CuFe_2_O_4_ and CuFe_2_O_4_–CQD was analyzed by nitrogen adsorption and desorption isotherm measurement. Figure S11a,b (Supporting Information) exhibit the CuFe_2_O_4_ surface area is 32.80 m^2^ g^−1^ and pore size diameter are 2.48 nm, Meanwhile, the surface area of CuFe_2_O_4_–CQD is 68.90 m^2^ g^−1^ (Figure S11c, Supporting Information) which is higher than CuFe_2_O_4_, whereas the pore size is smaller 1.95 nm (Figure S11d, Supporting Information). The carbon with the copper and iron ions can enhance the surface area of the CuFe_2_O_4_–CQD composite. This higher surface area and small pore size of CuFe_2_O_4_–CQD can improve the ion diffusion process in electrochemical reactions^[^ ^66^ ^]^ and enhance the dye molecules sorption ability in the dye degradation.

### 2.5 Supercapacitor Application

#### 2.5.1 Cyclic Voltammetry Study‐Three electrode System

**Figure 7:**
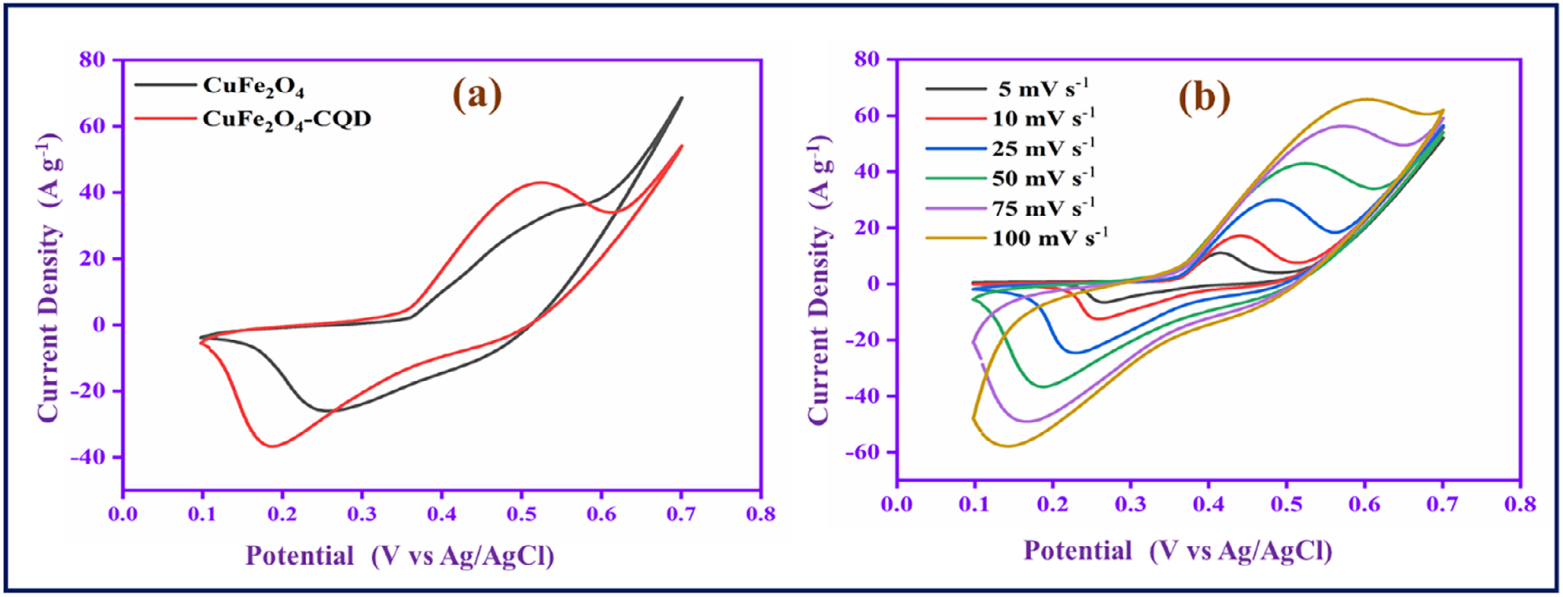
a) CV study of CuFe_2_O_4_ and CuFe_2_O_4_–CQD b) scan rate study of CuFe_2_O_4_–CQD.

The electrochemical behavior of CuFe_2_O_4_ and CuFe_2_O_4_–CQD materials was evaluated by cyclic voltammetry study. Understanding the pseudo‐capacitance nature of the materials involves a CV study for the potential range (0.1–0.7 V) in 1 m KOH, as shown in **Figure** **7**a. The CuFe_2_O_4_ electrode material redox pair peak presented at 0.25 and 0.50 V in the CV curve, exhibiting the rapid redox reaction of CuFe_2_O_4_ in the KOH electrolyte solution.

The redox reaction of CuFe_2_O_4_ in KOH can be presented as in Equation (1).

(1) $$\mathrm{CuF}e_{2}O_{4}+H_{2}O+2e^{-}⇆\mathrm{CuO}+2\mathrm{FeO}+2OH^{-}$$

The CuFe_2_O_4_–CQD redox pair potential is shifted and observed at 0.18 and 0.51 V due to the CQD present in the CuFe_2_O_4_ lattice structure. Moreover, the CuFe_2_O_4_–CQD (67 mA) has a higher range of peak current and larger CV curve area than CuFe_2_O_4_ (44 mA). The reason for the conductivity improvement might be the synergetic effect^[^ ^30^ ^]^ of CuFe_2_O_4_ and CQD, in which higher redox activity of CuFe_2_O_4_ is combined with electrical double‐layer capacitive CQD. The carbon material (CQD) can improve the electron transport during the electrochemical reaction process. Furthermore, the CuFe_2_O_4_–CQD involves a scan rate study (5–100 mV s^−1^). The scan rate affects the peak position, and the peak current is shown in Figure 7b. When the scan rate is increased, the CV area and current range linearly increase due to the fast‐redox process in the electrode‐electrolyte interfaces. The linear improvement of peak current suggests that the electrode‐electrolyte surface reaction is a diffusion‐controlled redox process. In a low scan rate, the diffusion layer covers the electrode surface, so the electrolyte flux toward the electrode is limited, and the result is a low current. But at a high scan rate, the diffusion layer cannot cover the electrode, so the electrolyte flux toward the electrode is enhanced, which helps to increase the current range. In the scan rate study, the upper and lower peaks are shifted toward the negative and positive directions, respectively, due to the development of overpotential. The overpotential can limit the faradic reaction.^[^ ^31^, ^67^ ^]^

#### 2.5.2 Capacitive and Diffusion‐Controlled Contributions Study

The capacitive and diffusion were studied to determine the capacitance ratio of the composite material. Figure S12a (Supporting Information) exhibits the diffusion and capacitive (shaded region) in the scan rate of 10 mV s^−1^. It shows 76% of surface capacitance and 24% of diffusion capacitance. Figure S12b (Supporting Information), the contribution rates of CuFe_2_O_4_–CQD composite from 5 to 100 mV s^−1^. The surface capacitance is increased and reaches 95% at a scan rate of 100 mV s^−1^ based on the scan rate improvement. The higher capacitive contribution confirms that the CuFe_2_O_4_–CQD has a fast reaction kinetics ability.^[^ ^32^ ^]^

#### 2.5.3 EIS Analysis

The electron and ions resistance behavior are studied by Electrochemical impedance spectroscopy analysis.^[^ ^68^ ^]^ The CuFe_2_O_4_ and CuFe_2_O_4_–CQD impedance spectra, as shown in Figure S13a,b (Supporting Information).

The equivalent circuits contain Warburg impedance element (W), constant phase element (CPE), charge transfer resistance (Rct), and solution resistance (Rs). The solution resistance (R_s_) value of CuFe_2_O_4_–CQD is lower (1.32Ω) than CuFe_2_O_4_ (1.07Ω), indicating the electrode‐electrolyte interface interaction is most favorable. The introduction of CQD in the CuFe_2_O_4_ matrix can improve the electron insertion–extraction process and increase the speed of ion transfer between the electrode and the electrolyte. The slope line at low frequency indicates the Warburg impedance. Compared to CuFe_2_O_4_, CuFe_2_O_4_–CQD has a short Warburg impedance and it is more vertical, which indicates the free ions movement in the electrolyte solution. These results demonstrated that the CuFe_2_O_4_–CQD ion diffusion resistance is less due to the synergetic effect that behavior enhances the electrical current and capacitance properties of the material. The CuFe_2_O_4_–CQD “n” value is 0.85, which suggests the electrode has pseudo supercapacitor behavior.^[^ ^69^ ^]^

#### 2.5.4 GCD Analysis

**Figure 8:**
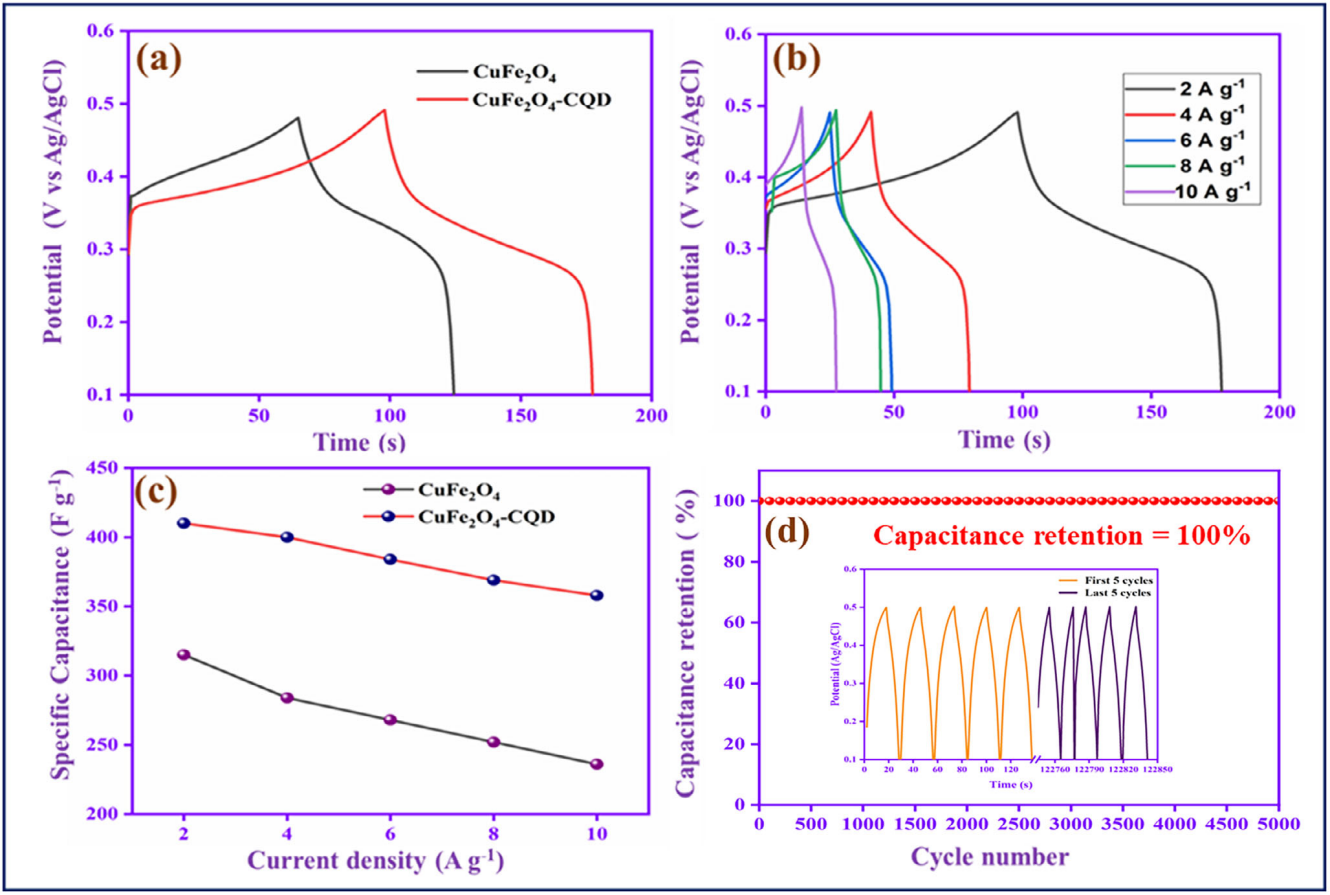
a) GCD study of CuFe_2_O_4_ and CuFe_2_O_4_–CQD, b) current density, c) specific capacitance value, and d) cycle stability analysis of CuFe_2_O_4_–CQD (Inset: First and last five cycles of GCD).

The GCD analysis of CuFe_2_O_4_ and CuFe_2_O_4_–CQD at 2 A g^−1^ current density is depicted in **Figure** **8**a. The discharge time of CuFe_2_O_4_–CQD is higher than CuFe_2_O_4_, confirming the good rate capability and electrochemical performance^[^ ^70^ ^]^ of the CuFe_2_O_4_–CQD. At current density 2 A g^−1^, the specific capacitance of CuFe_2_O_4_ is 315 F g^−1^ and CuFe_2_O_4_–CQD is 410 F g^−1^. The capacitance enhancement of CuFe_2_O_4_–CQD is possibly due to the composition of the electrical double‐layer capacitance performance of CQD and the pseudocapacitive nature of CuFe_2_O_4_.^[^ ^71^ ^]^ Figure 8b describes the different current density studies of CuFe_2_O_4_–CQD and Figure 8c shows the specific capacitance comparison CuFe_2_O_4_ and CuFe_2_O_4_–CQD at various current densities (2–10 Ag^−1^). Generally, a synergistic effect implies that the pairing of CuFe₂O₄ and CQDs leads to enhanced performance compared to either material on its own. Nevertheless, if the interaction between the two components is weak or if one material hinders the electrochemical activity of the other, such advantages may not be realized. Instead of a synergetic effect, charge storage behavior depends on the adsorption of electrolyte anions (OH^−^) on the metal oxide electrode. Typically, the charge storage capabilities of metal oxides, such as CuFe₂O₄, are significantly influenced by the extent to which electrolyte ions, particularly hydroxide ions (OH⁻), are absorbed onto their surface. These ions play a crucial role in redox reactions, which are responsible for the storage and release of charge. When the absorption of OH⁻ ions is lower, the number of redox reactions diminishes, resulting in reduced charge storage efficiency. Interestingly, a decrease in the insertion of OH⁻ ions into the electrode can lead to an increase in current density. This could occur because having fewer ions reduces internal resistance, allowing charges to move more rapidly and leading to a higher current despite lower storage efficiency. When the current density is increased, the rate of electrolyte anions (OH^−^) into the electrode is decreased. That is the reason the CuFe_2_O_4_ and CuFe_2_O_4_–CQD specific capacitance values are decreased (315‐ 236 Fg^−1^) and (410‐358 Fg^−1^) from 2 to 10 A g^−1^. The CuFe_2_O_4_–CQD kept 87.31% of its capacitance at the current density from 2 to 10 A g^−1^ while the CuFe_2_O_4_ has 74.92% of its capacitance. This result suggests that the CQD not only improved the capacitance but also enhanced the rate capability of CuFe_2_O_4_.^[^ ^72^ ^]^ Figure 8d shows the cyclic performance of the CuFe_2_O_4_–CQD electrode for 3000 charge–discharge cycles at 10 A g^−1^. After 5000 cycles, the electrode exhibits 100% capacitance retention. Table S4 (Supporting Information) shows the electrochemical performance comparison of CuFe_2_O_4_–CQD with reported CuFe_2_O_4_ and other metal oxides doped CQD. After cycle stability, the electrode involves structural analysis by XRD analysis, as shown in Figure S14 (Supporting Information). The CuFe_2_O_4_–CQD corresponding patterns (220), (311), (222), (400), (311), (511), and (440) appear before and after the cycle test electrodes. The XRD pattern shows no change after stability, whereas the peaks are slightly shifted due to the interaction electrolyte and binders in the electrode. The addition peaks correspond to the nickel foam substrate. The result concludes that the material is stable, which involves a reversible reaction on the surface during electrochemical reaction.

#### 2.5.5 Cyclic Voltammetry Analysis of Asymmetry (AC/PVA‐KOH/CuFe_2_O_4_–CQD) Supercapacitor Device

**Figure 9:**
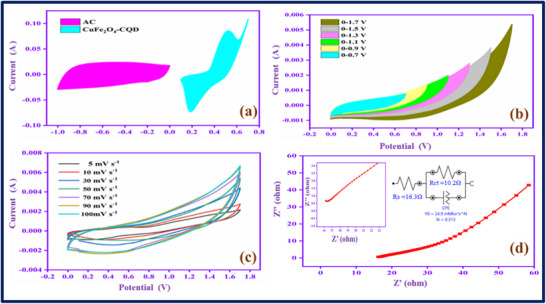
a) CV study of AC and CuFe_2_O_4_–CQD at 50 mV s^−1^ in 1 m KOH in three electrode systems, b) potential windows, c) scan rates, and d) EIS study of AC/PVA‐KOH/CuFe_2_O_4_–CQD asymmetric two electrode devices.

The three‐electrode system in the electrochemical setup can provide insight into the electrode material's electrochemical behavior for its supercapacitors applications. However, the two‐electrode device provides more significant results for commercial SCs applications. Therefore, we constructed an asymmetry supercapacitor (ASC) device, CuFe_2_O_4_–CQD as a cathode, activated carbon (AC) as an anode, and the PVA‐KOH as a gel electrolyte. The constructed device is AC/PVA‐KOH/CuFe_2_O_4_–CQD. **Figure** **9**a exhibits the CV profiles of AC and CuFe_2_O_4_–CQD electrodes at 50 mV s^−1^ in a three‐electrode setup, the working potential range is 0 to −1 and 0–0.7 V, respectively. The EDLC nature of AC and the pseudocapacitive nature of CuFe_2_O_4_–CQD are proved with the help of the shape of CV curves. Figure 9b displays the CV profile in different potential ranges at 50 mV s^−1^. The current and area of the CV curve gradually increase up to 1.7 V, which proves the potential window range of the ASC device is 0–1.7 V. So, the device involves different sweep rate studies from 5–100 mV s^−1^ in the range of 0–1.7 V, as depicted in Figure 9c. The CV current range is gradually increased with proper redox peaks presented in the curve profile up to 100 mV s^−1^, denoting the device has higher power capability at higher voltages. Figure 9d shows the electrochemical impedance spectroscopy (EIS), the plot has a vertical line in the low‐frequency region. The line represents the ideal behavior of the device owing to the speed of diffusion and adsorption in the electrode‐electrolyte interface.^[^ ^73^ ^]^ The solution resistance (Rs) value is 15.51Ω, demonstrating electrode‐electrolyte interface has rapid ion and electron transfer behavior.

#### 2.5.6 Galvanostatic Charge–Discharge Study of Asymmetry (AC/PVA‐KOH/CuFe_2_O_4_–CQD) Supercapacitor Device

The AC/PVA‐KOH/CuFe_2_O_4_–CQD device involves galvanostatic charge–discharge study, exhibited in Figure S15a (Supporting Information).

From the results, the predicted efficient potential window of AC/PVA‐KOH/CuFe_2_O_4_–CQD for the charge–discharge study is 0–1.5 V. The GCD curves have triangular shapes, indicating an excellent rate performance and electrochemical stability with a balanced charge storage mechanism.^[^ ^74^ ^]^ The device gives 277 F g^−1^ specific capacitance (Csp) at 2 A g^−1^ current density, shown in Figure S15b (Supporting Information). The Csp values gradually declined with increasing current density owing to their incomplete redox reaction in the electrode‐electrolyte interface and depressed ion penetration in the electrochemical reaction process. Energy and power density a pivotal parameters of supercapacitor applications that are calculated from the GCD measurements and correlated by Ragone plot,^[^ ^75^ ^]^ as shown in Figure S15c (Supporting Information). The device has 86 Wh kg^−1^ of energy and 1498 W kg^−1^ of power density at 2 A g^−1^. The energy density is still maintained at a higher current range (6 A g^−1^), the range is 23 Wh kg^−1^ (energy) and 4500 W kg^−1^ (power) density. Moreover, the device involves a galvanostatic cycle stability test as shown in Figure S15d (Supporting Information). The capacitance retention value of the device is 100% after repeating 5000 charge–discharge cycles. The result demonstrated that the AC/PVA‐KOH/CuFe_2_O_4_–CQD is a sufficient and potential device for commercial SCs applications

### 2.6 Photocatalytic Application

#### 2.6.1 UV–Vis Spectroscopy Study

Figure S16 (Supporting Information) demonstrates the methylene blue (MB) dye degradation study under visible light irradiation and the degradation analyzed by UV–vis spectroscopy. First, without a catalyst in MB had a 15% intensity loss after 120 min Figure S16a (Supporting Information) of visible light irradiation. Figure S16b (Supporting Information) illustrates the photocatalytic test of pure CuFe_2_O_4_ as a catalyst; the absorbance range of MB is gradually reduced based on the visible light irradiation time. With continuous visible light irradiation for 0–120 min, the MB absorption spectra have considerable intensity loss. After 120 min of irradiation, the absorbance is decreased by ≈55% from its initial stage. However, the CuFe_2_O_4_–CQD. Figure S16c (Supporting Information) shows the good MB degradation under visible light irradiation, with a 91% intensity loss after 120 min.

#### 2.6.2 Efficiency and Reusability Studies

**Figure 10:**
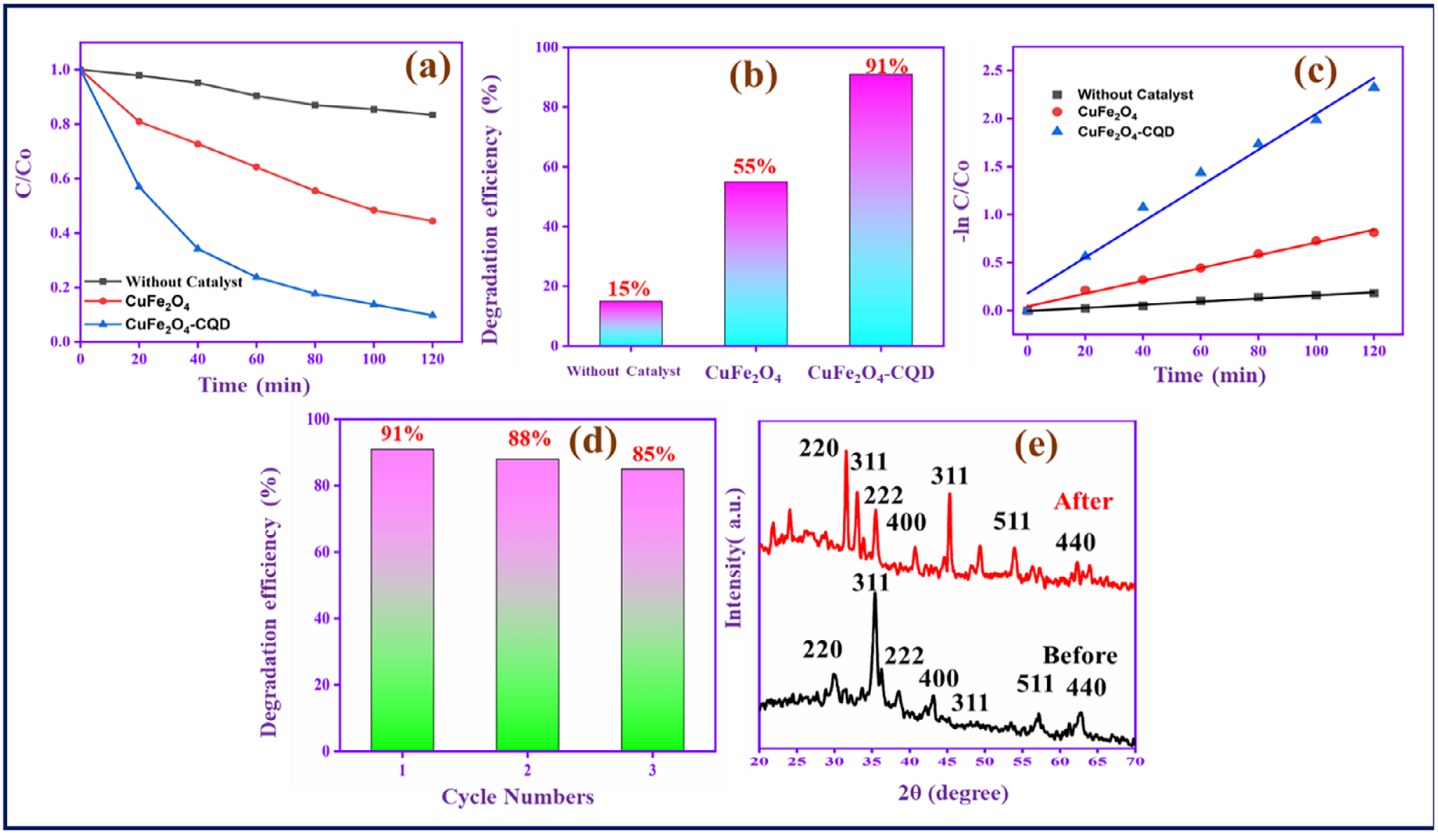
a) MB dye degradation rate, b) Different catalysts degradation efficiency, c) Rate constant study, d) Percentage of dye degradation in CuFe_2_O_4_–CQD based on reusability study, and e) XRD pattern of CuFe_2_O_4_–CQD based on reusability tests.

**Figure** **10**a,b shows the catalytic efficiency of dye degradation rates of CuFe_2_O_4_ and CuFe_2_O_4_–CQD in the presence of visible light. Interestingly, 91% of the dye is degraded when the CuFe_2_O_4_–CQD composite is used as a catalyst within 120 min. Meanwhile, without a catalyst and CuFe_2_O_4_ dye degradation efficiency is 15%, and 55%, respectively. The CuFe_2_O_4_–CQD composite has a synergistic effect between the CQD and CuFe_2_O_4_, which delivers a higher photocatalytic effect because which reduced recombination and enhancing charge transport.^[^ ^49^ ^]^ These e^−^/h^+^ separations can generate more active oxygen radicals like OH and O_2_^−^ from H_2_O and O_2_, which can easily degrade the dye molecules. Moreover, the CuFe_2_O_4_–CQD composite has a higher surface area, which helps to easily absorb the dye during the degradation process.^[^ ^76^ ^]^

Figure 10c exhibits the plot of the rate constant of MB dye degradation versus degradation time. The photocatalytic dye degradation reaction obeys the pseudo‐first‐order reaction kinetics, which is confirmed by the following equation ln(C/C_0_) = −kt, where k is the rate constant and t is the reaction time.^[^ ^77^ ^]^ The calculated k values for the MB dye degradation of CuFe_2_O_4_ are 0.0066739 min^−1^ and CuFe_2_O_4_–CQD is 0.0187 min^−1^. The CuFe_2_O_4_–CQD has a higher rate constant value than CuFe_2_O_4_, which indicates the CuFe_2_O_4_–CQD has good photocatalytic behavior. Moreover, stability and reusability of CuFe_2_O_4_–CQD is evaluability by the stability test in which the composite involves three cycles. Figure 10d shows only 6% dye degradation loss after three cycles. After the stability test, the material involves the structure or crystalline nature evaluation by XRD study as shown in Figure 10e. In this study, the XRD pattern crystal nature was not affected after cycle tests, indicating CuFe_2_O_4_–CQD has good reusability and higher chemical stability behavior.^[^ ^35^ ^]^

#### 2.6.3 Plausible Mechanism for Photodegradation of CuFe_2_O_4_–CQD

The bandgap analysis helps to understand the mechanism of the photocatalytic reaction. The calculated bandgap of CuFe_2_O_4_, CQD, and CuFe_2_O_4_–CQD are 1.82, 3.10, and 1.80 eV, as depicted in Figure S17 (Supporting Information). The calculated electronegativity of the CuFe_2_O_4_, CQD, and CuFe_2_O_4_–CQD is 5.8,7.5, and 6.85 eV, respectively. Figure S18 (Supporting Information) demonstrates the energy level diagram of the CuFe_2_O_4_, CQD, and CuFe_2_O_4_–CQD composite. The E_CB_ and E_VB_ values are calculated by the χ and E_g_ (Table S5, Supporting Information).

Figure S19 (Supporting Information) shows the dye degradation mechanism between CuFe_2_O_4_ and CQD. In the presence of visible light irradiation, the CuFe_2_O_4_ and CQD absorb the photon in the CuFe_2_O_4_–CQD composite. In the first step, CuFe_2_O_4_ absorbs the visible light, excites the electron from the VB to CB, and produces an electron (e^−^) in the CB while leaving the holes (h^+^) in the VB. The CB electron of CuFe_2_O_4_ can transfer to the CQD CB, whereas the CQD also involves an excitation process and generates electrons and holes in the CB and VB band, respectively. The electrons react with H_2_O and generate O_2_− and OH radicals. And in the oxidation process, the holes react with H_2_O and produce. OH radicals. These radicals easily degrade the MB, in which the radicals break down the dye molecules' aromatic bonds and convert them into smaller molecules like CO_2_ and H_2_O.^[^ ^78^ ^]^ Comparison analysis of CuFe_2_O_4_–CQD with other reported devices is shown in Table S6 (Supporting Information).

The photocatalytic dye degradation process of the MB mechanism steps in the presence of CuFe_2_O_4_–CQD under visible light are shown in Equations (2), (3), (4), (5), (6).^[^ ^79^ ^]^

(2) $$\begin{array}{ccc} \mathrm{CuF}e_{2}O_{4}/\mathrm{CQD}+hv & \to & \mathrm{CuF}e_{2}O_{4}\;\left({e^{-}}_{\left(\mathrm{CB}\right)}+{h^{+}}_{\left(\mathrm{VB}\right)}\right)/\\ & & \mathrm{CQD}\;\left({e^{-}}_{\left(\mathrm{CB}\right)}+{h^{+}}_{\left(\mathrm{VB}\right)}\right) \end{array}$$

(3) $$\begin{array}{ccc} \mathrm{CuF}e_{2}O_{4}\;\left({e^{-}}_{\left(\mathrm{CB}\right)}\right)+\mathrm{CQD}\;\left({e^{-}}_{\left(\mathrm{CB}\right)}\right) & \to & \left({e^{-}}_{\left(\mathrm{CB}\right)}\right)+\mathrm{CQD}\;\left(e_{\left(\mathrm{CB}\right)}^{-}\right)\\ & & +\;\mathrm{CuF}e_{2}O_{4} \end{array}$$

(4) $$O_{2}+\left({e^{-}}_{\left(\mathrm{CB}\right)}\right)+\mathrm{CQD}\;\left({e^{-}}_{\left(\mathrm{CB}\right)}\right)\to O_{2^{\cdot -}}+\mathrm{CQD}$$

(5) $$\mathrm{CQD}\;\left({h^{+}}_{\left(\mathrm{VB}\right)}\right)+H_{2}O\to H^{+}+\cdot \mathrm{OH}+\mathrm{CQD}$$

(6) $$\begin{array}{ccc} O_{2^{\cdot -}} & + & \cdot \mathrm{OH}+C_{16}H_{18}CN_{3}\mathrm{SCl}\;\left(\mathrm{MB}\right)\to \\ & & CO_{2}+H_{2}O+\mathrm{degraded}\;\mathrm{products} \end{array}$$

## 3 Conclusion

The preparation of CQD from ascorbic acid and their properties, such as absorption and emission behavior, functional groups, structural properties, and surface charge of CQD were evaluated. The selectivity and sensitivity of the metal ion study of CQD are studied, which can detect Fe^3+^ and Cu^2+^ ions through a fluorometric technique. The LOD of metal ions is determined by a titration profile study, the values are CQD‐Fe^3+^–0.36 µM and CQD‐Cu^2+^–0.59 µM. Moreover, the cytotoxicity and the fluorescence imaging behavior of CQD were analyzed in MDA‐MB‐231 cells. Due to the higher binding ability of CQD with Fe^3+^ and Cu^2+^ ions, the CuFe_2_O_4_–CQD composite material is prepared by the hydrothermal method. The CuFe_2_O_4_–CQD delivers 410 F g^−1^ capacitance, and the dye degradation efficiency is 91% in 120 min. The overall result concludes that the understanding performance of CQD and the CuFe_2_O_4_–CQD in the field of energy and environmental applications highlights the future development and utilization of CQD in commercial applications. Researchers can develop the usage and CQD and their composite toward practical applications, which may reduce the energy demand and global warming issues.

## 4 Experimental Section

### Materials

Ascorbic acid and ethanol were purchased from Sisco Research Laboratories Pvt. Ltd (SRL). All metal salts were obtained from Himedia Chemicals. Solutions of Fe^2+^, Fe^3+^, Cu^2+^, Co^2+^, Al^3+^, Ba^2+^, Mg^2+^, Mn^2+^ and Pb^2+^ were prepared from their nitrate salts.

### Preparation of CQD

2 g of ascorbic acid was dissolved in 30 mL ethanol: water (1:1) and stirred for 1 h at room temperature. Then, the transparent solution was transferred into a 100 mL autoclave and heated at 180 °C for 10 h. Then, the brown color solution was treated and extracted with dichloromethane. Finally, the solution involved a dialysis process for 2 days, then stored at room temperature for further use.^[^ ^19^ ^]^

### Preparation of CuFe_2_O_4_–CQD

20 mmol of copper nitrate and 40 mmol of ferric nitrate (1:2) were dissolved in 50 mL of water. Then, 5 mL of CQD was added to the solution. The yellow color was changed to brownish–red when the CQD was added. The color change indicates that the CQD reduced the metal ions and produced a CQD‐doped metal ion nanocomposite. Then, the solution was transferred to the autoclave and heated at 180 °C for 6 h. Finally, the solution was calcined at 300 °C for 3 h to obtain the copper ferrite –CQD (CuFe_2_O_4_–CQD) composite, as shown in Scheme S1 (Supporting Information), For comparison, the same method was followed to produce the CuFe_2_O_4_ without the addition of CQD.^[^ ^80^ ^]^

### Metal Sensing Analysis of CQD using Fluorescence Spectroscopy

The 1 mL of CQD was diluted with 100 mL of distilled water, which acts as a fluorescence probe. The metal nitrate salts such as Al^3+^, Ba^2+^, Fe^3+^, Fe^2+^, Co^2+^, Cu^2+^, Mg^2+^, Mn^2+^, and Pb^2+^ in 10^−6^ m were dissolved in distilled water in the 10 mL standard measuring flask. The 2 mL of diluted CQD was taken in a quartz cuvette, and 50 µM of each metal was added to the solution for analysis of the metal detection behavior of CQD by fluorescence spectroscopy.^[^ ^15^ ^]^

### Sensitivity and Selectivity Study

A sensitivity study helps to analyze the binding stoichiometric of metal‐probe interactions. The fluorescence behavior was determined by adding the stock solution of selective ions (0–50 µM) in 2 mL of CQD during titration. The change in the probe fluorescence intensity based on the metal concentration improvement was shown by the concentration of metal ions versus fluorescence emission. The limit of detection (LOD) was determined by FL spectra data. A linear graph was plotted and fitted.^[^ ^81^ ^]^ The plotted curve uses to LOD value from Equation 7.

(7) $$\mathrm{LOD}=3.3\left(\mathrm{Sy}/S\right)$$

In which Sy denotes the response of standard deviation and S is the slope of the linear curve.

The selectivity study was examined by interference analysis. The common ion interference was measured in fluorescence after the addition of 2 mL of CQD with selective metal ions. The CQD‐selective metal ions complexes were exposed to the addition of each metal ion. After 5 min, the emission behavior was measured.

### Real Sample Analysis

Water samples were collected from different water resources, such as tap, pond, and sea, and spiked with known concentrations of selective metal ions to prepare the real sample pattern for analysis. The sample was filtered using a syringe filter to remove the contaminated particles from the water. Then, the analytical test was done three times, and the common statistics were reported.

### Density Functional Theory (DFT) Study

The Density functional theory (DFT) of CQD, CQD–Fe^3+^, and CQD–Cu^2+^ was carried out by Gaussian 16. The structure of CQD was constructed and optimized. The study is based on the B3LYP exchange‐correlation function with a 6–31+G (d,p) basis set.^[^ ^82^ ^]^ The molecule modeling method was given the optimized structure and minimum energy of CQD, CQD–Fe^3+^, and CQD–Cu^2+^ and the energy values of the highest occupied molecular orbitals (HOMO) and lowest unoccupied molecular orbital (LUMO).

### Cell Viability Test of CQD

Human breast cancer (MDA‐MB‐231) cells were collected from the National Center for Cell Sciences (NCCS), Pune, India. It was maintained in Dulbecco's Modified Eagle medium (DMEM). For imaging studies, cells were seeded in 96‐well plates and incubated for 24 h.^[^ ^83^ ^]^ After the incubation period, the cells were treated with various concentrations of CQD for 24 h. Then, the cells were washed with PBS, and the new medium, Cell Counting Kit‐8, into the cells. After 4 h of incubation, the absorbance range of CQD was measured with the help of a microplate reader for the calculation of cell viability. The experimental procedure was repeated four times to calculate the statistical analysis.

### Cell Culture and Fluorescence Imaging

The MDA‐MB‐231 cells were seeded on a 35 mm cell culture dish in which 2× 10^5^ cells per dish were cultured in DMEM medium for 24 h. The cells were treated with 1 µL mL^−1^ CQD for 4 h in DMEM medium and washed with PBS three times. After washing, the cells were stained with Hoechst for 10 min. Then, the images were captured with the help of a confocal microscope.

### Fabrication of Asymmetry Supercapacitor Device (AC/PVA‐KOH/ CuFe_2_O_4_–CQD)

In a two‐electrode system, the activated carbon (AC) is a negative electrode, the CuFe_2_O_4_–CQD is a positive electrode, and the PVA‐KOH is a gel electrolyte. The two electrodes were separated by the cellulose filter paper, which acts as a separator. First, 1.5 g PVA was added to 1 m KOH aqueous solution, and then, the mixture was heated at 70 °C with vigorous stirring until a transparent gel solution was formed. Before fabrication, the electrodes were soaked in the gel electrolyte and dried. Then, the two electrodes were placed with the PVA‐KOH gel electrolyte and wrapped with a plastic film. The fabricated supercapacitor device (AC /PVA‐KOH/ CuFe_2_O_4_–CQD) involves electrochemical analysis.

### Physical and Electrochemical Characterization, Photocatalytic Activity, and Bandgap Calculation

This section is provided in the Supporting Information.

### Ethical Approval

No humans or animals have been used in this research

## Conflict of Interest

The authors declare no conflict of interest.

## Author Contributions

E.S. performed conceptualization methodology, formal analysis, and wrote the original draft. A.R., N.K.K., K.V., and M.K.M. performed data curation and visualization. N.S. provided funding, wrote, reviewed, and edited the final manuscript. S.T. performed supervision, wrote, reviewed, and edited the final manuscript.

## Supporting information



Supporting Information

## Data Availability

The datasets used or analyzed during the current study are available from the corresponding author upon reasonable request.
