# Supplementary material for: Preparation and Characterization of Carbon Quantum Dots (CQD) and CuFe2O4–CQD Composite Materials for Photo and Electrochemical Applications
Source: Glob Chall. 2025 Jun 9;9(7):e00044. doi: 10.1002/gch2.202500044 (PMC12246727; doi:10.1002/gch2.202500044)
Supplement: Supplementary file 1 — Supporting Information [file GCH2-9-e00044-s001.docx]

**PREPARATION AND CHARACTERIZATION OF CARBON QUANTUM DOTS(CQD) AND CuFe_2_O_4_-CQD** **COMPOSITE MATERIALS FOR PHOTO AND ELECTROCHEMICAL APPLICATIONS**

**Esakkimuthu Shanmugasundaram^1^, Amos Ravi^2^, Nithesh Kumar Krishnan^1^, Kannan Vellaisamy^1^, Murali Krishnan Mani^3^, Na’il Saleh^4^*, Stalin Thambusamy^1^***

^1^Department of Industrial Chemistry, Alagappa University, Karaikudi - 630 003, Tamil Nadu, India**.**

^2^Department of Materials Science, Central University of Tamil Nadu, Thiruvarur - 610 005,

Tamil Nadu, India.

^3^Department of Chemistry, Bannari Amman Institute of Technology, Sathyamangalam,

Erode - 638 401, Tamil Nadu, India.

^4^Department of Chemistry, College of Science, United Arab Emirates University, P.O. Box 15551, Al Ain, United Arab Emirates.

*Corresponding author e-mail: ***Stalin Thambusamy -** *[stalin.t@alagappauniversity.ac.in](mailto:stalin.t@alagappauniversity.ac.in)., ***Na’il Saleh -** [n.saleh@uaeu.ac.ae](mailto:n.saleh@uaeu.ac.ae).


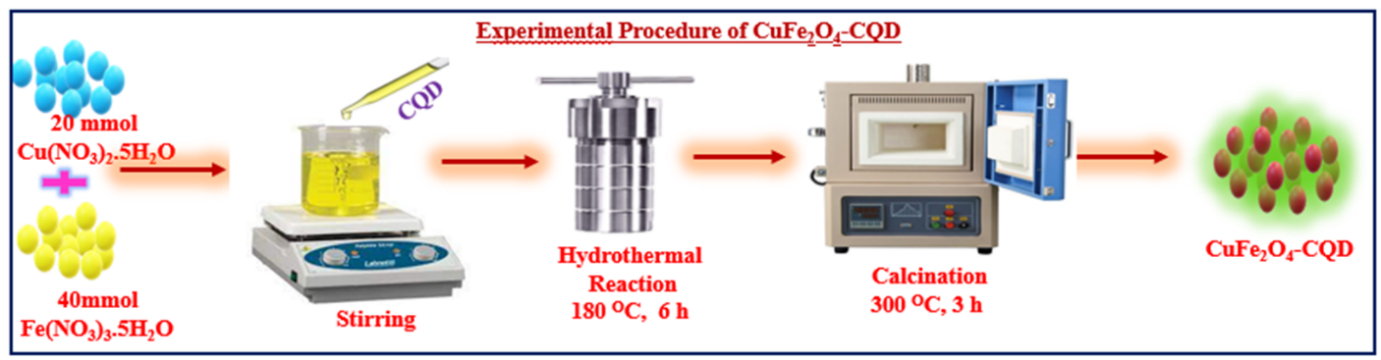


**Scheme 1. The preparation procedure of CuFe_2_O_4_-CQD**

**1. Characterizations**

**1.1. Physical Characterization:** The absorption behavior of the samples is analyzed by JASCO NIS 670 (liquid) UV-visible spectrophotometer (Range-200 to 800 nm). The functional group is evaluated by JASCO 4600 Fourier transform infrared spectrometer (FT-IR) (Range - 400-4000 cm^-1^). The phase and crystal structure are investigated by X-ray diffraction spectrometer-XRD (XRD - X’ Pert Pro – PANalytic; source - Cu Kα). The functional group was also analyzed with the help of the Raman Spectrometer - Confocal Raman Microscope. The element composition is investigated by X-ray photoelectron spectroscopy (X’ Pert Pro – PAnalytic). The surface morphology was analyzed by scanning electron microscopy (SEM, NOVA NANO 450) equipped with energy dispersive X-ray analysis (EDAX) and transmission electron microscope (TEM, FEI Tecnai G2 20 S-TWIN). The porous nature and surface area were examined by Brunauer-Emmett-Teller (BET) techniques using the Nova 2200e model. The electrochemical characterization was analyzed by Meterohm Autolab (NOVA software).

**1.2. Electrochemical analysis:** The electrochemical studies were studied by metrohm electrochemical analyzer MULTIAUTOLAB-M204 (NOVA software) with three electrodes set up Ni foam coated prepared materials (4 mg prepared sample + 0.5 mg polyvinylidene fluoride + 0.5 mg activated carbon) act as a working electrode, Ag/AgCl as a reference electrode and platinum wire (1 × 1 surface area), as a counter electrode. The electrochemical characterization such as cyclic voltammetry (CV), electrochemical impedance spectroscopy (EIS) and Galvanostatic charge-discharge (GCD) was done by 1M KOH electrolyte. The electrochemical impedance spectroscopy (EIS) was investigated in the frequency range of 0.01 Hz -100 kHz, the amplitude is 10 mV at open circuit voltage.

In a two-electrode system, the activated carbon (AC) is a negative electrode, the CuFe_2_O_4_ - CQD is a positive electrode and the PVA-KOH is a gel electrolyte. The two electrodes are separated by the cellulose filter paper which acts as a separator.

The specific capacitance (Cs) in equation 1, Energy density (E) in equation 2 and power density (P) in equation 3 of the prepared electrode materials was calculated by the below-given formula is

Cs = I × ∆t / ∆v× m (1)

Where, Cs - specific capacitance, I - discharge current, ∆t-discharge time and m - mass of active material.

E = [ C × (∆v)2]/7.2 (2)

P = E × 3600/∆t (3)

P - power density and E - energy density, C - specific capacitance, X ∆t - voltage and ∆t - discharge time.

**1.3. Photocatalytic activity test:** The photocatalytic dye degradation of methylene blue (MB) was conducted by visible light irradiation in a photochemical reactor. In each experiment, photocatalyst (50mg) was added in 100 mL of an aqueous solution of MB (0.0319 mg/mL). The prepared catalyst dye suspensions are stirred in the dark for 30 minutes before the experiment to achieve the adsorption/desorption equilibrium. After that, the catalyst suspensions were exposed to visible light (60 mW/cm) for 120 minutes. Every 10 minutes, MB solution (5mL) was taken and centrifuged to remove the solid catalyst. Then, the solution concentration was analyzed using UV-Vis spectrometry.

The percentage of degradation was calculated by the following (equation 4|),

Degradation (%) = 100 x$\frac{C_{0}-C_{t}}{C_{0}}$ (4)

$C_{0}$ -Initial and $C_{t}$ (t=min) concentration of dye molecule, the catalyst involves cycle tests to analyze its stability and reusability. The catalyst was used for three cycles in the photocatalytic experimental tests. In each cycle, the catalyst was collected and centrifuged. The collected samples were dried at 60 ^o^C and used in next cyclic process.

**1.4. Band gap calculation:** The band gap of the photocatalytic materials is calculated by Tauc plot (equation 5)

α (hυ) = A* (hυ - Eg) n/2 (5)

α -absorption coefficient, h - Planck's constant, t - light frequency, A - constant's value, Eg - bandgap energy, and n - number of transitions in the semiconductor. The formula mentioned above is used to compute the bandgap

Moreover, the valence band (VB) and conductance band (CB) potential ranges are calculated using the following (equation 6-8)

E_CB_ = χ – Ec − 0.5Eg ` (6)

E_VB_ = E_CB_ + Eg (7)

χ = [χ Aa χ Bb χ Cc]1/(a+b+c) (8)

where E_CB_ and E_VB_ are edge potentials, Eg is the band gap of the semiconductor material, Ec is the energy of free electrons on the hydrogen scale (4.5 eV), χ represents the Mulliken electronegativity and a, b, and c represent the number of atoms in the compound.


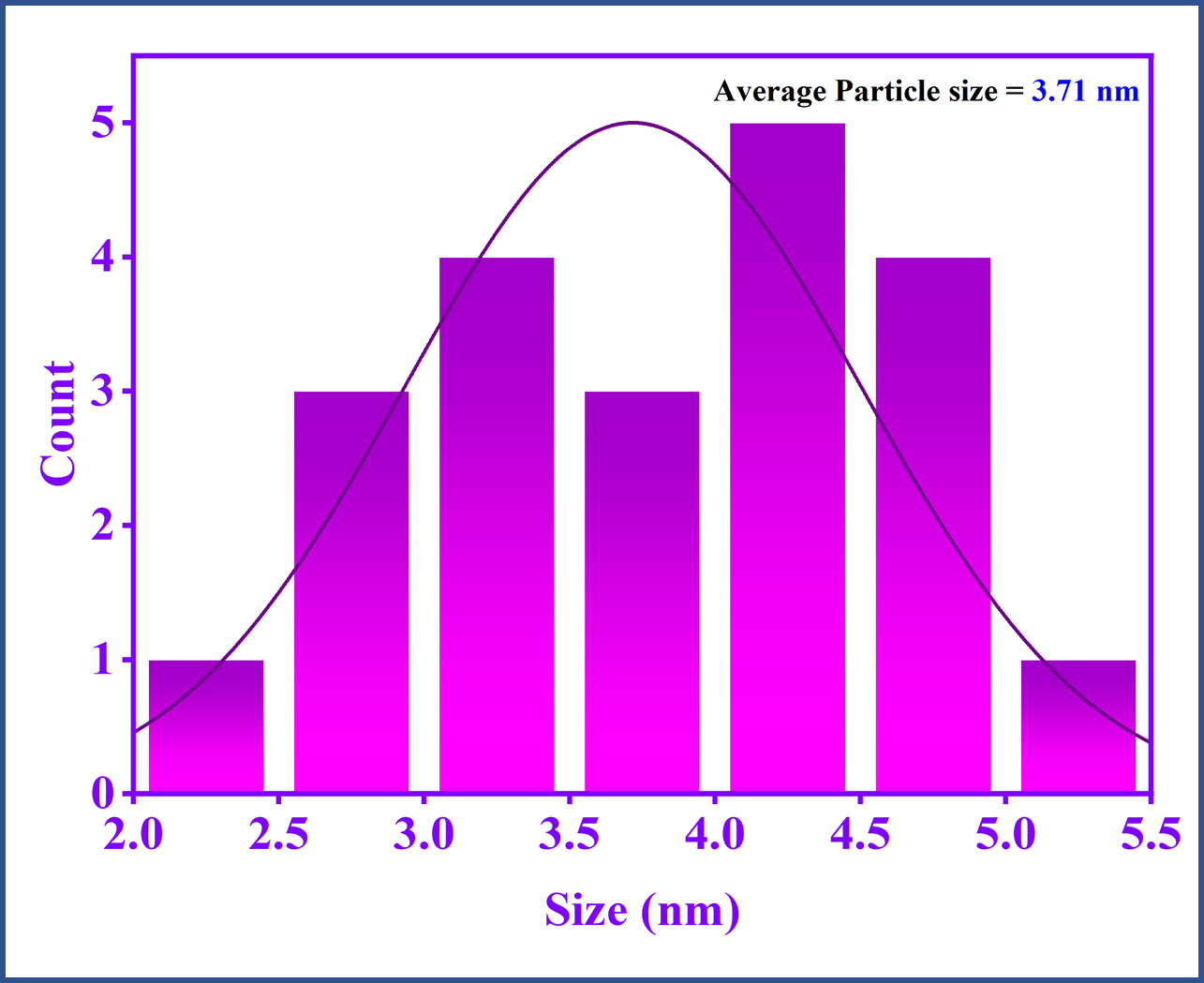


**Figure S1.** Histogram of the particle size distribution of CQD.


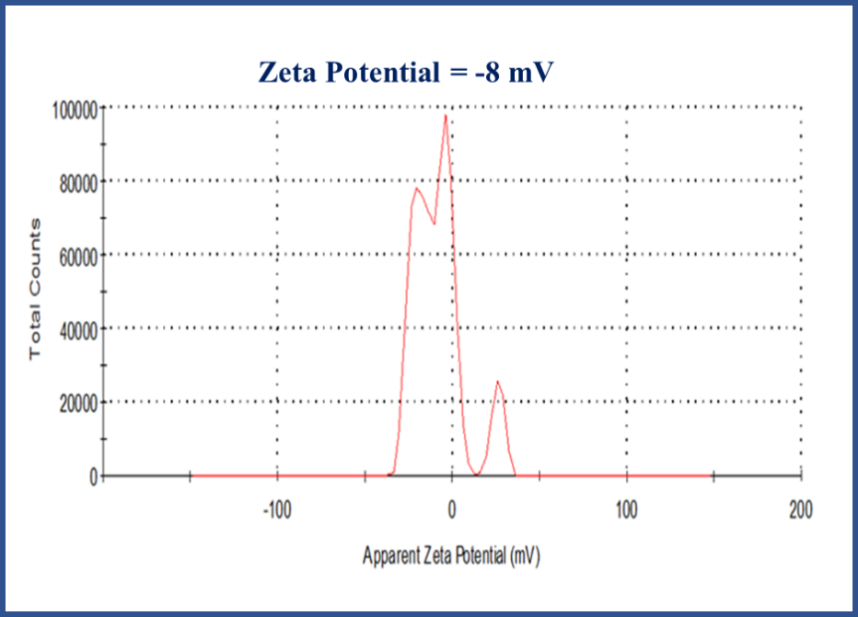


**Figure S2.** Zeta Potential study of CQD.

**
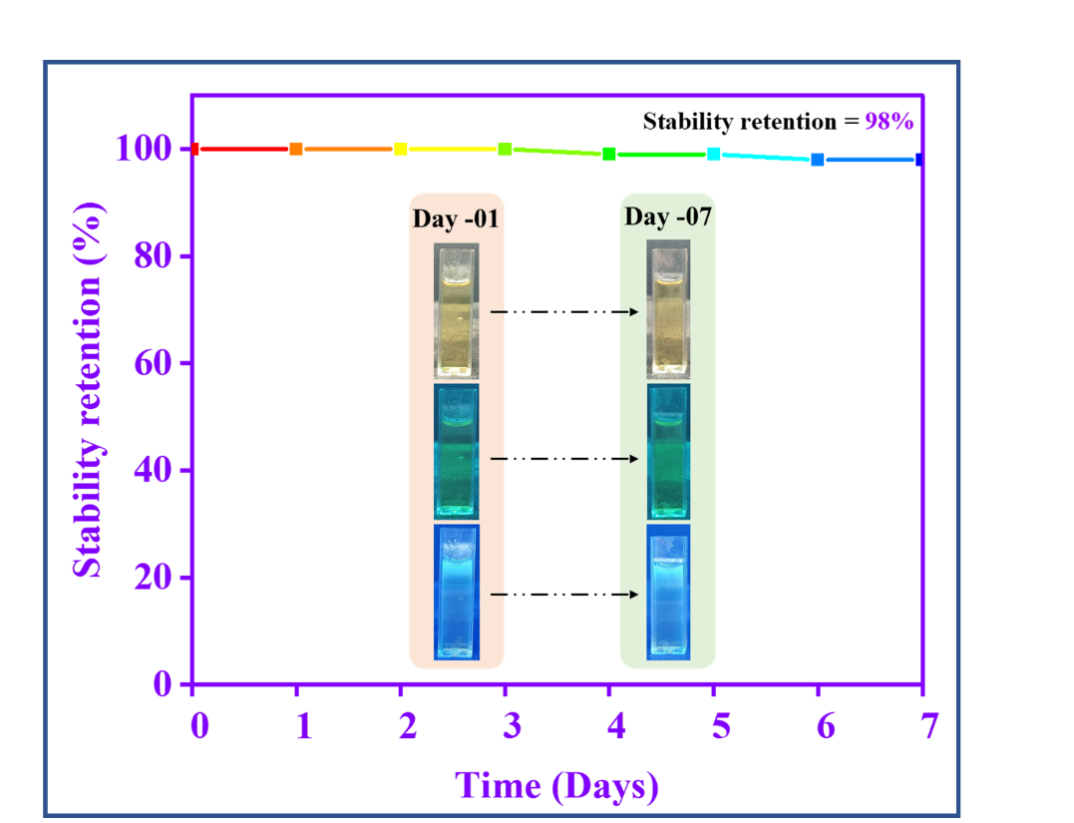
**

**Figure S3.** Stability study of CQD.

**
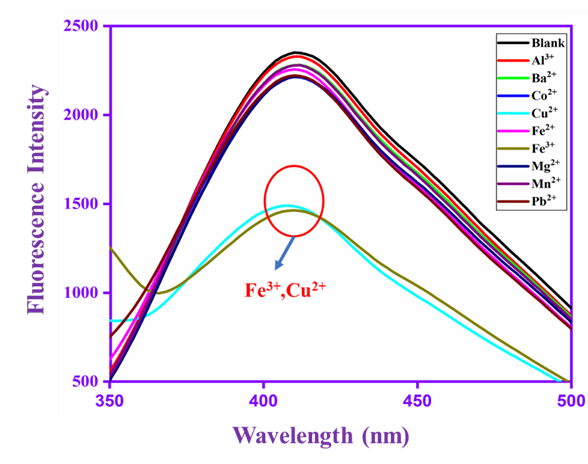
**

**Figure S4.** Fluorescence spectral response of CQD with different metal ions.


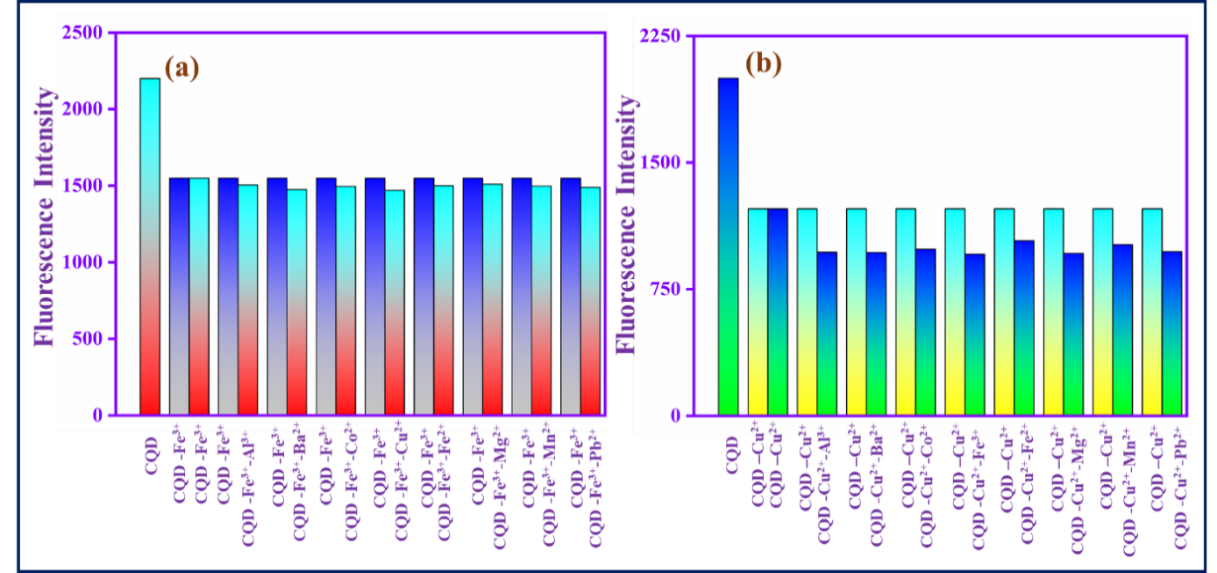


**Figure S5.** Interference bar diagram of fluorescence intensities of various metal ions mixed with (a) CQD - Fe^3+^ and (b) CQD - Cu^2+^.

**Table S1.** Determination of Fe^3+^ concentrations in different real water samples.

| Probe | Type of real sample | Added  species | Spiked  *(μM*) | Found  *(μM*) | Recovery  (%) |
| --- | --- | --- | --- | --- | --- |
| CQD | Tap Water | Fe^3+^ | **0**  **5**  **10** | 0  4.96 ± 0.003  9.82 ± 0.004 | 0  99.2  98.2 |
|  | Pond Water | Fe^3+^ | **0**  **5**  **10** | 0  4.86 ± 0.010  9.20 ± 0.003 | 0  97.2  92.0 |
|  | Sea water | Fe^3+^ | **0**  **5**  **10** | 0  4.86 ± 0.015  9.80 ± 0.003 | 0  97.2  98.0 |

**Table S2.** Determination of Cu^2+^ concentrations in different real water samples.

| Probe | Type of real sample | Added  species | Spiked  *(μM*) | Found  *(μM*) | Recovery  (%) |
| --- | --- | --- | --- | --- | --- |
| CQD | Tap Water | Cu^2+^ | **0**  **5**  **10** | 0  4.71 ± 0.002  9.62 ± 0.003 | 0  95.2  96.2 |
|  | Pond Water | Cu^2+^ | **0**  **5**  **10** | 0  4.85 ± 0.010  9.40 ± 0.003 | 0  94.8  94.0 |
|  | Sea water | Cu^2+^ | **0**  **5**  **10** | 0  4.76 ± 0.015  9.78 ± 0.003 | 0  96.2  94.9 |

**Table S3.** Metal sensing performance of CQD in comparison with other reported CQD

| **S. No.** | **Source** | **Selectivity** | **Limit of detection** | **Ref** |
| --- | --- | --- | --- | --- |
| 1 | sodium citrate and urea | Hg^2+^ | 0.65 µM | [1] |
| 2 | D-glucose and ethane-1,2- diamine | Cu^2+^ | 1.8 µM | [2] |
| 3 | Peanut shells | Cu^2+^ | 4.8 µM | [3] |
| 4 | Whey | Se^2+^ | 0.78 µM | [4] |
| 5 | Macauba fibres | Fe^3+^ | 0.99 μM | [5] |
| 6 | Lignocellulose-based CQD | Fe^3+^ | 62.5 μM | [6] |
| 7 | Magnolia flower | Fe^3+^ | 0.073 μM | [7] |
| **8** | Lignin | Fe^3+^ | 0.77 μM. | [8] |
| **9** | **CQD** | **Cu^2+^**  **Fe^3+^** | **0.59 µM**  **0.36 µM** | **This work** |


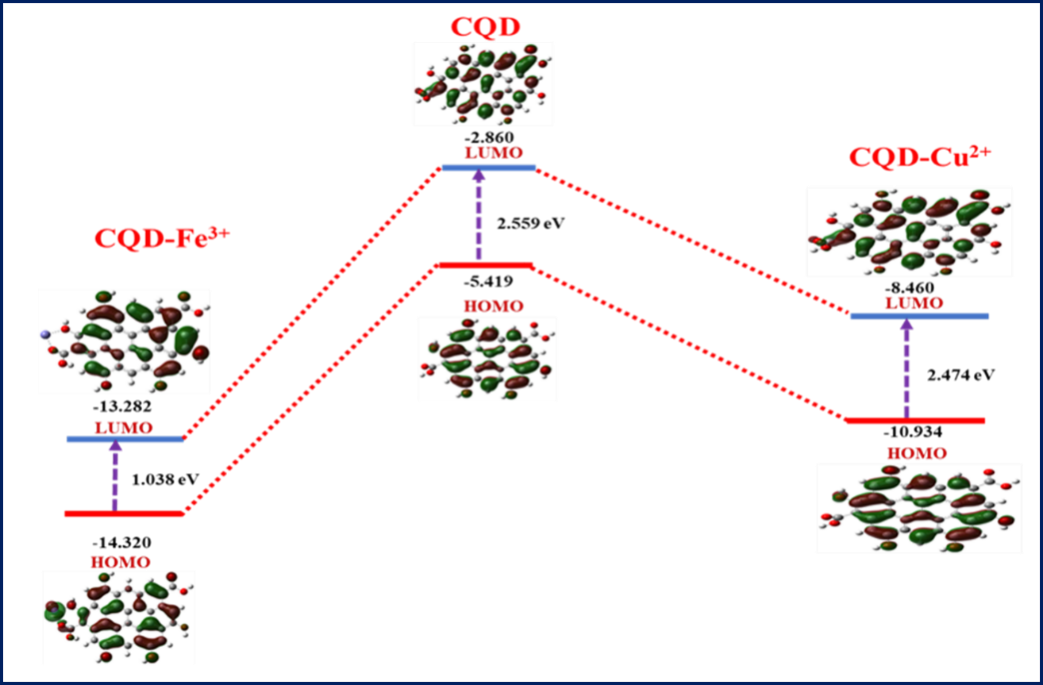


**Figure S6.** Density functional theory study of CQD, CQD - Fe^3+^, and CQD - Cu^2+^.


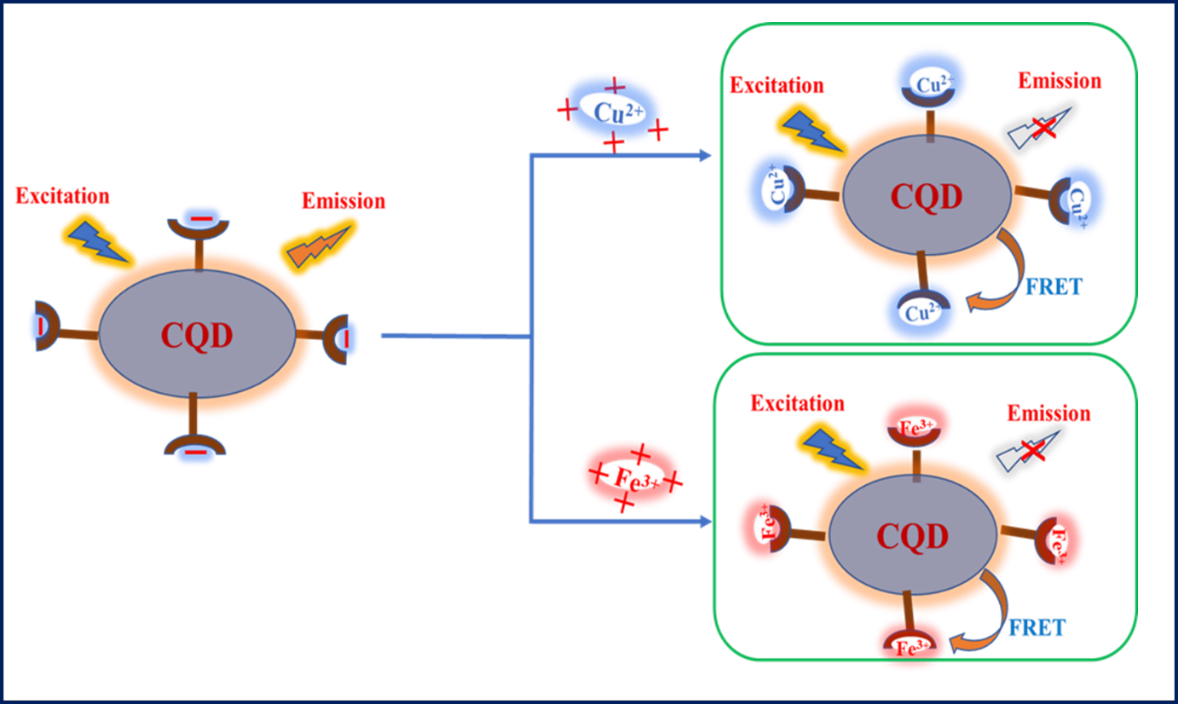


**Figure S7.** Plausible FRET transfer mechanism of CQD, CQD-Fe^3+,^ and CQD –Cu^2+^.


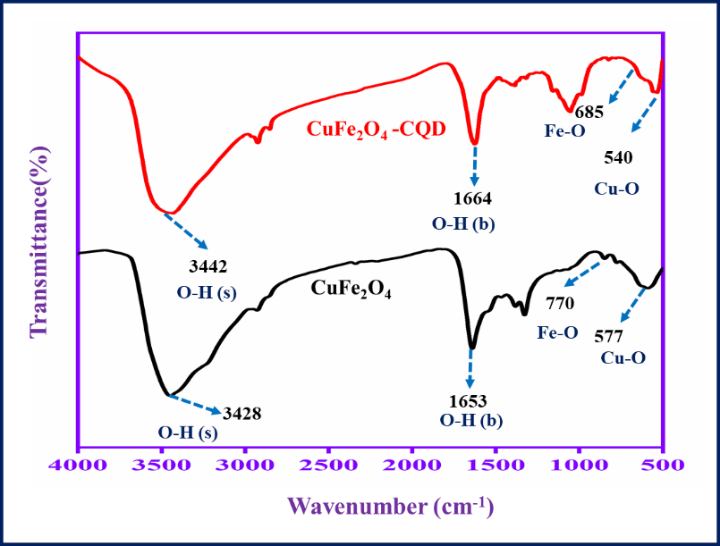


**Figure S8.** FT-IR spectra of CuFe_2_O_4_ and CuFe_2_O_4_-CQD.


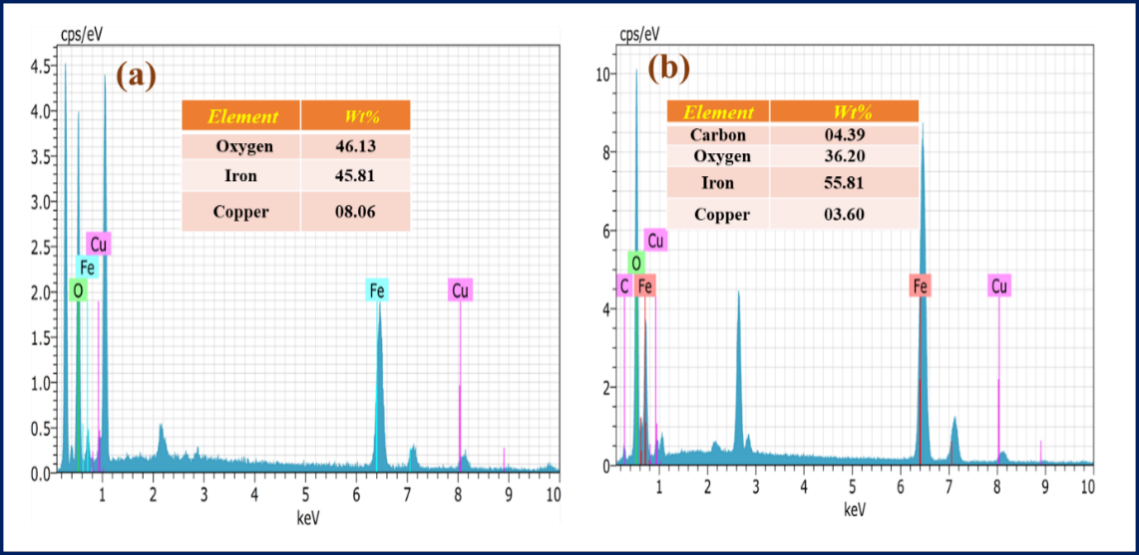


**Figure S9.** EDX analysis of (a) CuFe_2_O_4_ and (b) CuFe_2_O_4_-CQD.


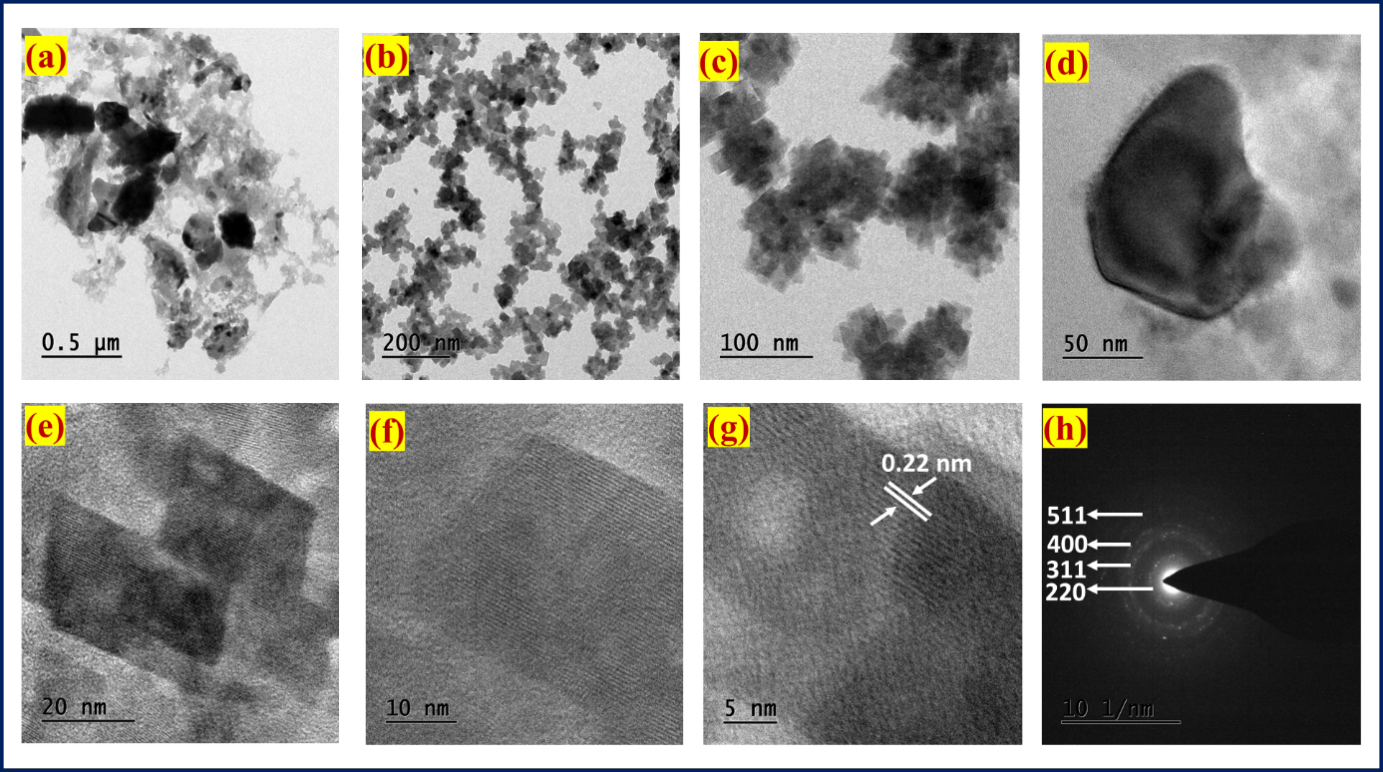


**Figure S10.** TEM images of (a-g) CuFe_2_O_4_ - CQD in different magnifications, and (d) SAED patterns of CuFe_2_O_4_ - CQD.


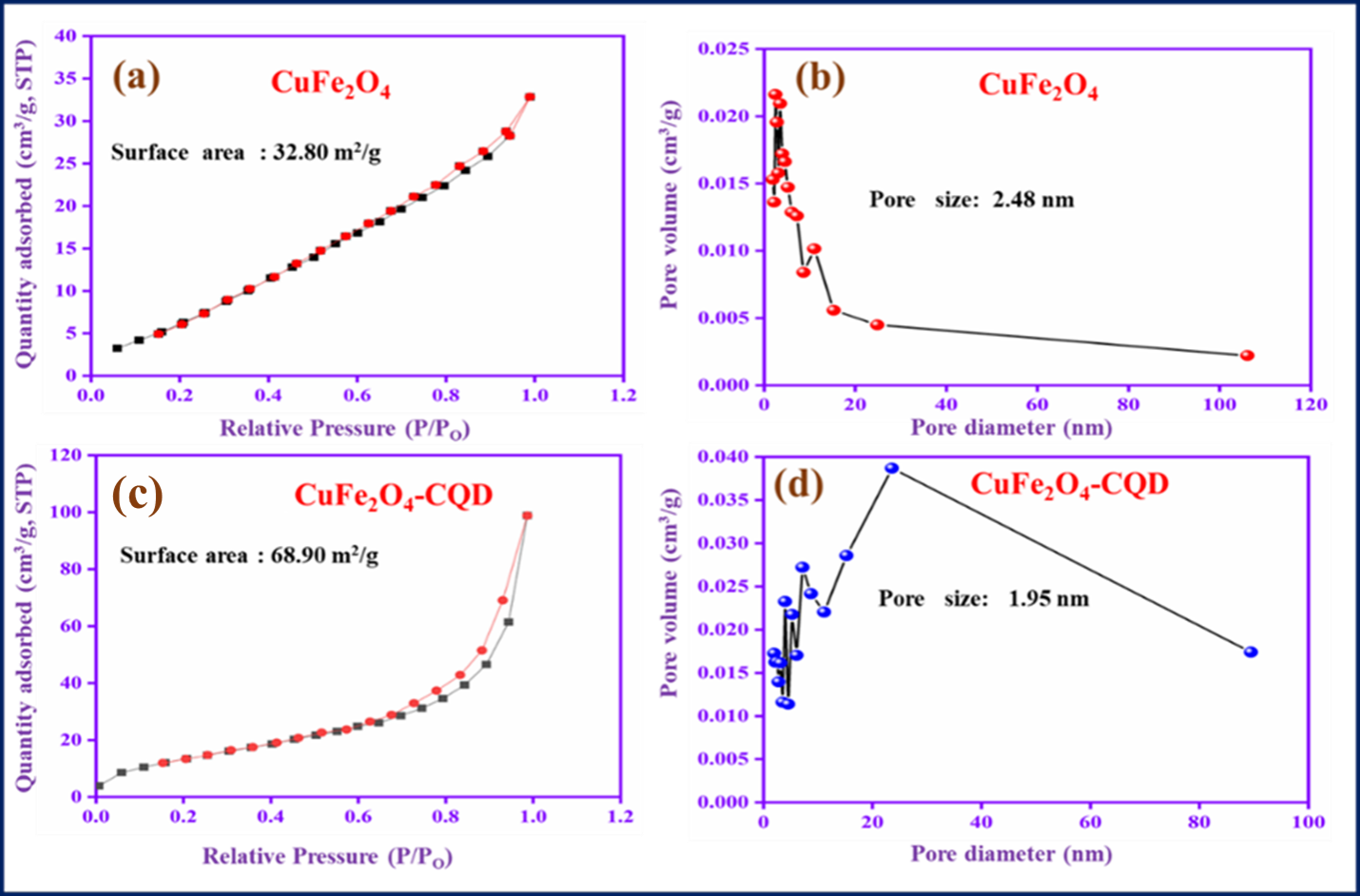


**Figure S11.** Nitrogen adsorption-desorption isotherms for (a) CuFe_2_O_4_ and Pore size distributions of (b) CuFe_2_O_4_; Nitrogen adsorption-desorption isotherms for (c) CuFe_2_O_4_-CQD, and (d) Pore size distributions of CuFe_2_O_4_-CQD.

**
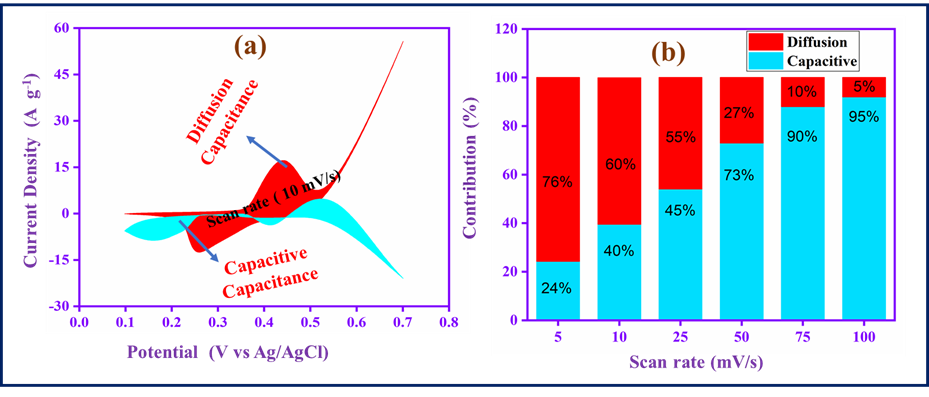
**

**Figure S12.** (a) The proportion of capacitive and diffusion capacitance for charge storage contributions of CuFe_2_O_4_ - CQD at 10 mV/s (b) Comparison of the stored charge at scan rates of 5,10,25,50,75 and 100 mV/s.


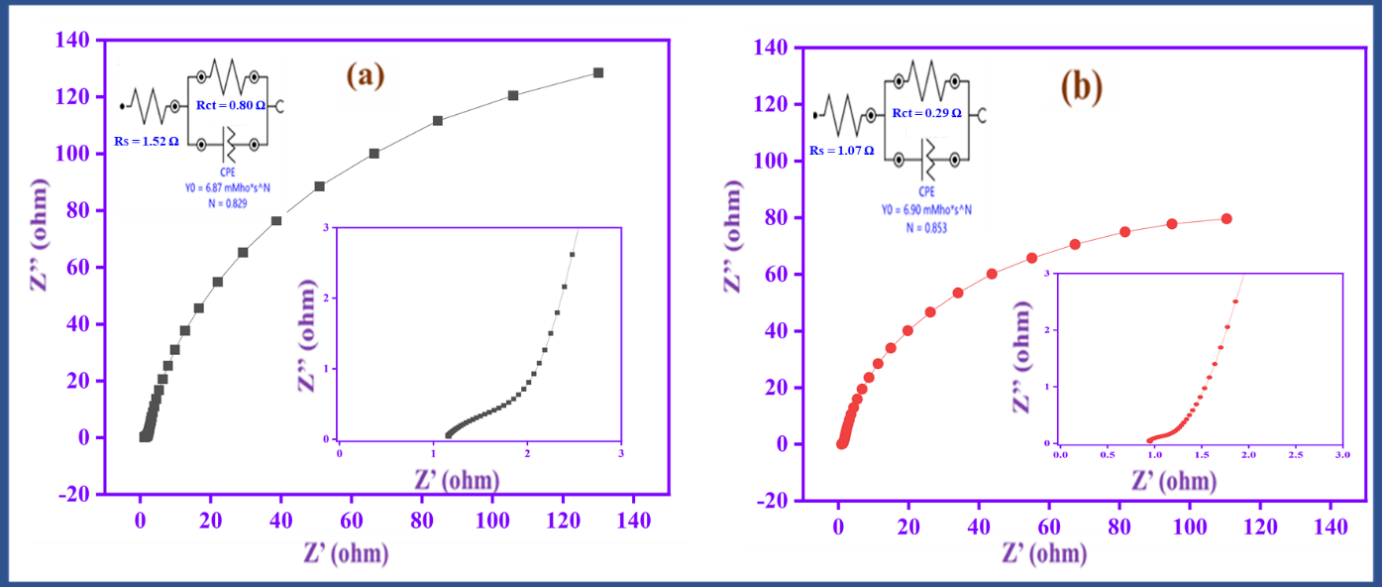


**Figure S13.** EIS spectra and the equivalent circuits of (a) CuFe_2_O_4_ (b) CuFe_2_O_4_ – CQD (Inset: High-frequency region).

**Table S4.** Electrochemical performance of CuFe_2_O_4_-CQD in comparison with reported CuFe_2_O_4_ and other metal oxides doped CQD-based materials

| **S. No.** | **Electrode material** | **Capacitance** | **Cycle stability** | **Ref** |
| --- | --- | --- | --- | --- |
| 1 | CQD- MnO_2_ | 189 at 0.14 A g^−1^ | 100%, (1200) | [9] |
| 2 | CQD−Bi_2_O_3_ | 343 at 0.5 A g^−1^ | 95%, (2500) | [10] |
| 3 | CQDs- MnO_2_ | 340 at 1 A g^−1^ | 80%, (10000) | [11] |
| 4 | MoS_2_/NCDs | 149.21 at 0.5 A g^−1^ | 100%, (2000) | [12] |
| 5 | CuMnO_2_/GQD | 520.2 at 0.5 A g^−1^ | 83.3% (5000) | [13] |
| 6 | CuFe_2_O_4_ | 189.2 at 0.5 A g^−1^ | 84% (1000) | [14] |
| 7 | CuFe_2_O_4_-GN | 576 F g^-1^ at 2 A g^-1^ | 85% (1000) | [15] |
| S8 | CuFe_2_O_4_-rGO | 313 F g^-1^ at 2 A g^-1^ | - | [16] |
| **9** | **CuFe_2_O_4_-CQD** | **410 F g^-1^ at 2 A g^-1^** | **100%, (3000)** | **This work** |


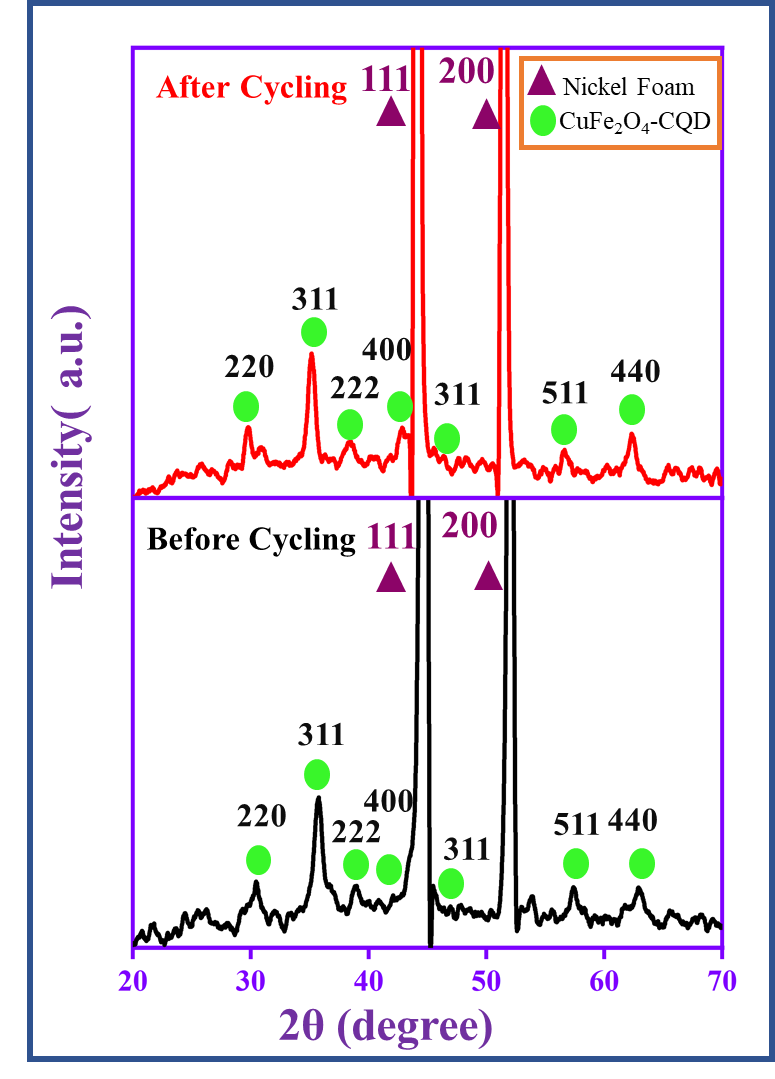


**Figure S14. XRD spectra of before and after cyclic stability study of CuFe_2_O_4_-CQD.**


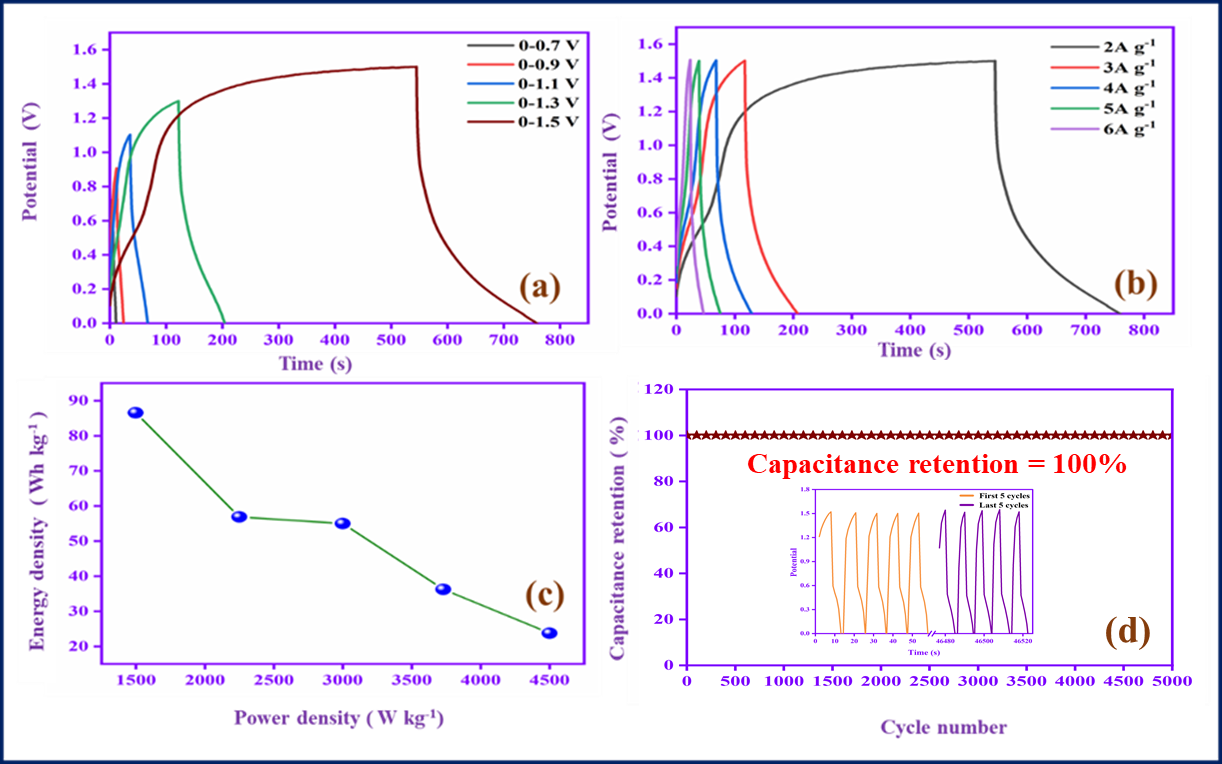


**Figure S15.** (a) Different voltage GCD, (b) Different current density GCD, (c) Ragone diagram and, (d) Cycle stability studies of AC/PVA-KOH/CuFe_2_O_4_-CQD asymmetric two electrode devices (Inset: First and last five cycles of GCD).


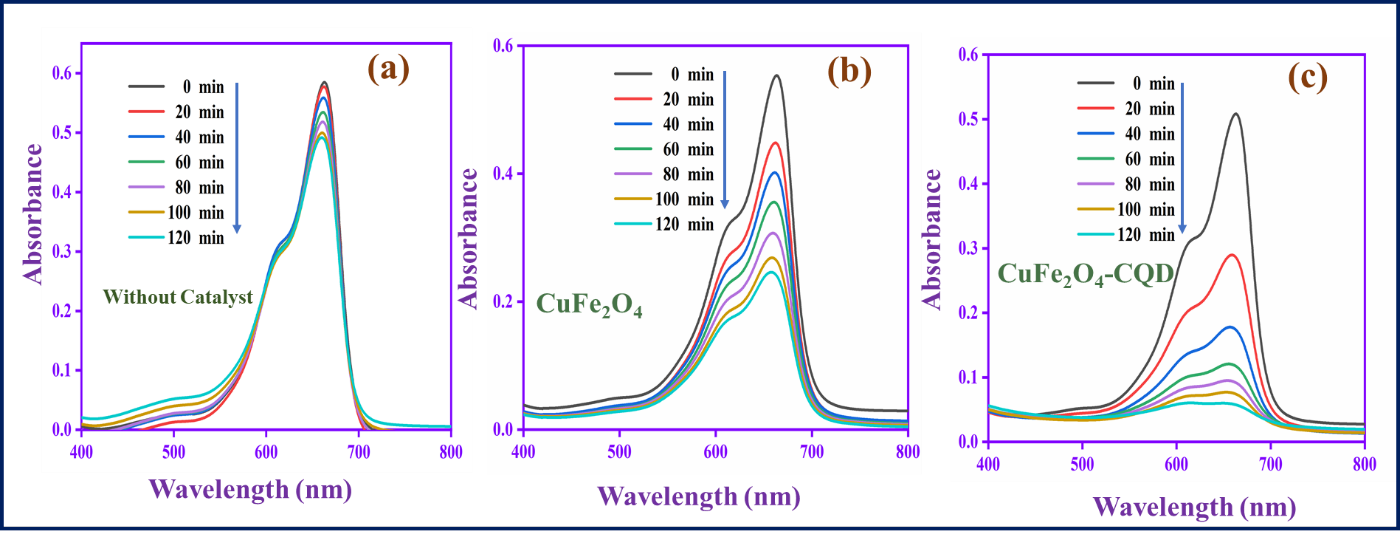
 **Figure S16.** Photocatalytic performance of (a) Without Catalyst of MB, (b) CuFe_2_O_4_, and (c) CuFe_2_O_4_-CQD under visible light irradiation for about 120 min.


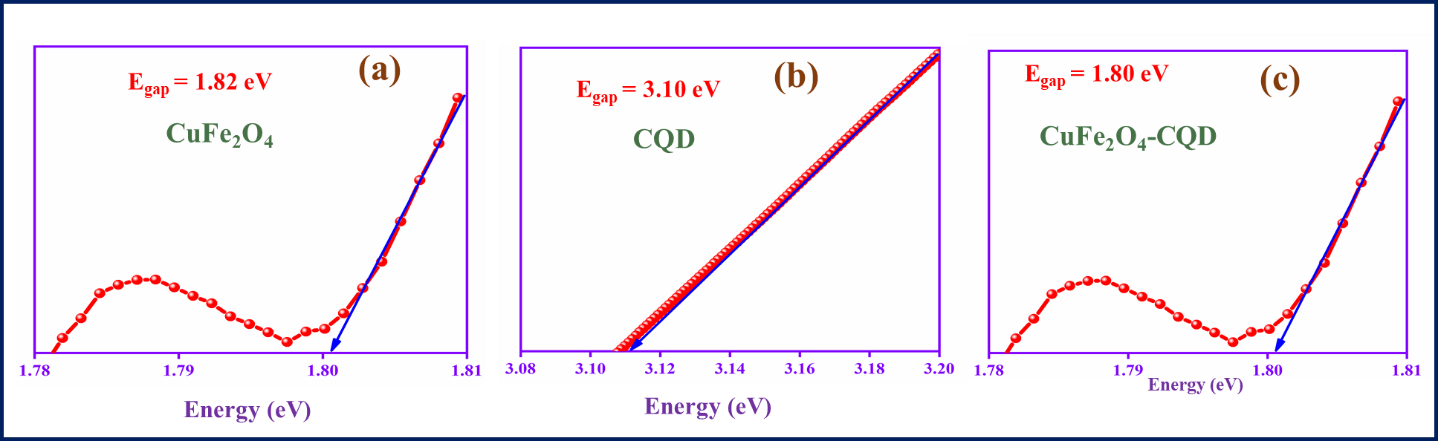


**Figure S17.** Band gap study of (a) CuFe_2_O_4_, (b) CQD, and (c) CuFe_2_O_4_-CQD composite.


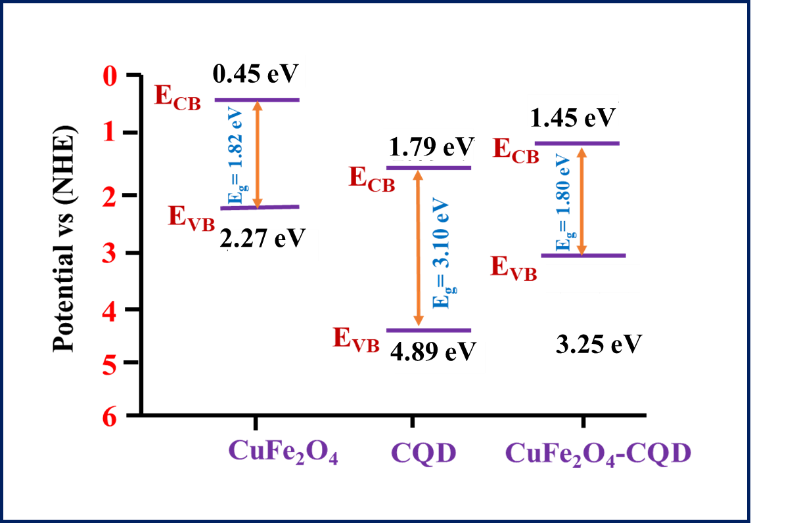


**Figure S18.** Band gap structure of CuFe_2_O_4_, CQD, and CuFe_2_O_4_-CQD.

**Table S5.** The Values of the Bandgap (E_g_), Conduction Band (E_CB_), and valence band (E_VB_) Edge Potentials

| **Materials** | **E_g_ (eV)** | **E_CB_ (eV)** | **E_VB_ (eV)** |
| --- | --- | --- | --- |
| CuFe_2_O_4_ | 1.82 | 0.45 | 2.27 |
| CQD | 3.10 | 1.79 | 4.89 |
| CuFe_2_O_4_-CQD | 1.80 | 1.45 | 3.25 |


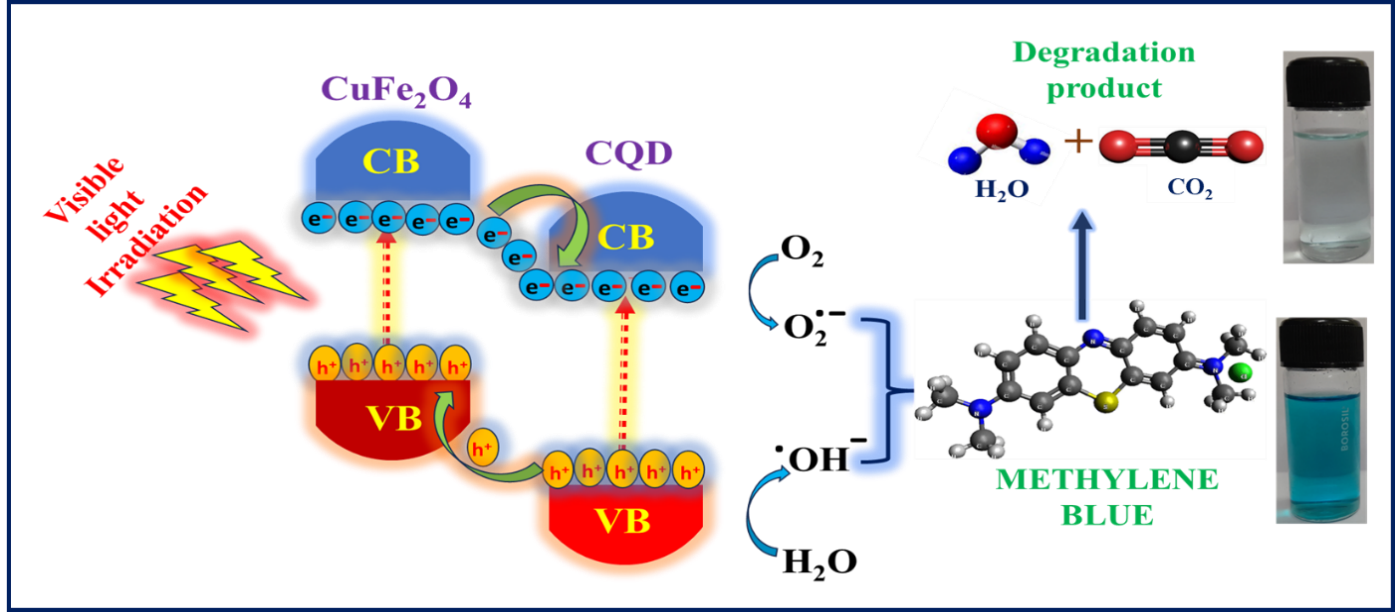


**Figure S19.** Schematic diagram of dye degradation and electron charge transfer mechanism of CuFe_2_O_4_-CQD underss visible light irradiation.

**Table S6.** Comparison analysis of CuFe_2_O_4_-CQD with other reported devices.

| S.No | Catalyst | Pollutant | Degradation (%) | Ref. |
| --- | --- | --- | --- | --- |
| 1. | CuFe_2_O_4_ | Methylene blue | 84.3 % | [17] |
| 2. | CuFe_2_O_4_/g-C_3_N_4_ | Methylene blue | 82 % | [18] |
| 3 | N-ZnO/CD | Methylene blue | 58.2% | [19] |
| 4 | TiO_2_/CQD |  | 89.9% | [20] |
| 5. | **CuFe_2_O_4_-CQD** | **Methylene blue** | **91 %**  **in 120 min** | **Present work** |

**References**

[1] G. Ren, Y. Meng, Q. Zhang, M. Tang, B. Zhu, F. Chai, C. Wang, Z. Su, *New J. Chem.* **2018**, *42*, 6824–6830.

[2] V. Singh, V. Kumar, U. Yadav, R. Kr. Srivastava, V. N. Singh, A. Banerjee, S. Chakraborty, A. K. Shukla, D. K. Misra, R. Ahuja, A. Srivastava, P. S. Saxena, *ISSS J. Micro Smart Syst.* **2017**, *6*, 109–117.

[3] X. Ma, Y. Dong, H. Sun, N. Chen, *Mater. Today Chem.* **2017**, *5*, 1–10.

[4] P. Devi, G. Kaur, A. Thakur, N. Kaur, A. Grewal, P. Kumar, *Talanta* **2017**, *170*, 49–55.

[5] S. Raja, G. T. S. T. Da Silva, S. Anbu, C. Ribeiro, L. H. C. Mattoso, *Biomass Conv. Bioref.* **2024**, *14*, 21925–21937.

[6] Y. Qiu, D. Li, Y. Li, X. Ma, J. Li, *Cellulose* **2022**, *29*, 367–378.

[7] C. Wang, H. Shi, M. Yang, Y. Yan, E. Liu, Z. Ji, J. Fan, *Mater. Res. Bull.* **2020**, *124*, 110730.

[8] L. Zhu, D. Shen, Q. Liu, C. Wu, S. Gu, *Appl. Surf. Sci.* **2021**, *565*, 150526.

[9] A. Prasath, M. Athika, E. Duraisamy, A. S. Sharma, P. Elumalai, *ChemistrySelect* **2018**, *3*, Article 30.

[10] A. Prasath, M. Athika, E. Duraisamy, A. S. Sharma, V. Sankar Devi, P. Elumalai, *ACS Omega* **2019**, *4*, 3.

[11] H. Lv, X. Gao, Q. Xu, H. Liu, Y. G. Wang, Y. Xia, *ACS Appl. Mater. Interfaces* **2017**, *9*, 46.

[12] H. M. El Sharkawy, A. S. Dhmees, A. R. Tamman, S. M. El Sabagh, R. M. Aboushahba, N. K. Allam, *J. Energy Storage* **2020**, *27*.

[13] M. Ashourdan, A. Semnani, F. Hasanpour, S. E. Moosavifard, *J. Energy Storage* **2021**, *36*.

[14] Y. Guo, Y. Chen, X. Hu, Y. Yao, Z. Li, *Colloids Surf. A Physicochem. Eng. Asp.* **2021**, *631*.

[15] W. Zhang, B. Quan, C. Lee, S. K. Park, X. Li, E. Choi, G. Diao, Y. Piao, *ACS Appl. Mater. Interfaces* **2015**, *7*, 4.

[16] P. Makkar, D. Gogoi, D. Roy, N. N. Ghosh, *ACS Omega* **2021**, *6*, 43.

[17] S. Munir, A. Rasheed, S. Zulfiqar, M. Aadil, P. O. Agboola, I. Shakir, M. F. Warsi, *Ceram. Int.* **2020**, *46*, 29182–29190.

[18] R. Li, M. Cai, Z. Xie, Q. Zhang, Y. Zeng, H. Liu, G. Liu, W. Lv, *Appl. Catal. B Environ.* **2019**, *244*, 974–982.

[19] D. G. Ayu, S. Gea, Andriayani, D. J. Telaumbanua, A. F. R. Piliang, M. Harahap, Z. Yen, R. Goei, A. I. Y. Tok, *ACS Omega* **2023**, *8*, 14965–14984.

[20] M. Shafique, M. S. Mahr, M. Yaseen, H. N. Bhatti, *Mater. Chem. Phys.* **2022**, *278*, 125583.
